# Temporal transcriptome analysis of neuronal commitment reveals the preeminent role of the divergent lncRNA biotype and a critical candidate gene during differentiation

**DOI:** 10.1038/s41420-020-0263-6

**Published:** 2020-04-24

**Authors:** Bharat Prajapati, Mahar Fatima, Mena Fatma, Priya Maddhesiya, Himali Arora, Teesta Naskar, Subhashree Devasenapathy, Pankaj Seth, Subrata Sinha

**Affiliations:** 1 National Brain Research Centre, Manesar, Gurgaon, Haryana India; 2 Department of Biochemistry, All India Institute of Medical Sciences, New Delhi, 110029 India

**Keywords:** Molecular neuroscience, Neural stem cells

## Abstract

lncRNA genes can be genic or “intergenic”. “Genic” RNAs can be further divided into six biotypes. Through genome-wide analysis of a publicly available data set on corticogenesis, we found that the divergent lncRNA (XH) biotype, comprising the lncRNA and the coding gene being in opposite directions in a head-to-head manner, was most prominent during neural commitment. Within this biotype, a coding gene/divergent RNA pair of the BASP1 gene and the uncharacterized RNA loc285696 (hitherto referred as BASP1-AS1) formed a major HUB gene during neuronal differentiation. Experimental validation during the in vitro differentiation of human neural progenitor cells (hNPCs) showed that BASP1-AS1 regulates the expression of its adjacent coding gene, BASP1. Both transcripts increased sharply on the first day of neuronal differentiation of hNPCs, to fall steadily thereafter, reaching very low levels in differentiated neurons. BASP1-AS1 RNA and the BASP1 gene formed a molecular complex that also included the transcription factor TCF12. TCF12 is coded by the DYX1 locus, associated with inherited dyslexia and neurodevelopmental defects. Knockdown of BASP1-AS1, BASP1, or TCF12 impaired the neuronal differentiation of hNPCs, as seen by reduction in DCX and TUJ1-positive cells and by reduced neurite length. There was also increased cell proliferation. A common set of critical genes was affected by the three molecules in the complex. Our study thus identified the role of the XH biotype and a novel mediator of neuronal differentiation—the complex of BASP1-AS1, BASP1, and TCF12. It also linked a neuronal differentiation pathway to inherited dyslexia.

## Introduction

Long noncoding RNAs (lncRNAs) are emerging as key regulators of coding genes^1–4^. LncRNA transcripts have a low coding potential and are more than 200 ribonucleotides long. The number of lncRNAs in the human genome surpasses the number of coding genes. The brain, the most specialized organ in the body, comprises a diversity of cell types, with a complex structural and functional organization. In total, 40% of all lncRNAs are expressed in distinct brain regions^2,5^. The functions of lncRNAs in pluripotency and neuronal differentiation^6,7^ are now emerging.

lncRNAs^1,8^ are annotated on the basis of their genomic position with respect to the protein-coding genes^9^. According to such a classification, lncRNA biotypes broadly fall into two types—genic lncRNA (<5 kb to a coding gene) and intergenic IG. Genic lncRNAs are further categorized into six biotypes: divergent or antisense head-to-head (XH), convergent or antisense tail-to-tail (XT), antisense outside (XO), antisense inside (XI), sense downstream (SD), and sense upstream (SU). Studies point to the prominent role of divergent lncRNAs (XH) during development and cell fate determination^9–12^. Of late, there has been an exponential growth in high-quality annotated data available for public access. Analysis of such data helps in identifying the possible functions of lncRNA biotypes, followed by the role of individual genes. Accompanied by experimental validation, it could lead to the identification of novel cellular pathways, including neuronal differentiation.

The “CORTECON” database^13^ provides an atlas of mRNA expression of the in vitro development of the cerebral cortex from human embryonic stem cells (ESCs). This is from the ESC stage (day 0) to day 77, when the morphological organization and markers of the cortex are very evident. We have analyzed the lncRNA-associated coding genes of this database with respect to markers. Based on expression of markers, day 0 was taken as equivalent to ESCs, and day 7 as equivalent of human neural progenitor cells (hNPCs). Subsequent stages were indicated by markers of differentiated neurons. While there could be lack of synchrony in the in vitro cortex differentiation model between cells and various stages, the predominant markers were taken to be representative of major cell types ranging from ESCs, hNPCs, to differentiated neurons. Based on different forms of clustering analyses, we were able to identify a major association of the XH lncRNA biotypes with the neuronal commitment stage. Within this, by using algorithm for the reconstruction of accurate cellular networks (ARACNe) and hierarchal clustering, we have been able identify the BASP1 gene as a major HUB gene during neuronal differentiation (day 7). BASP1 is the coding partner of an uncharacterized XH lncRNA gene (loc285696, hitherto referred as BASP1-AS1).

This prompted us to study the role of the BASP1/BASP1-AS1 pair in a model where human fetal-derived hNPCs were differentiated into neurons under defined conditions in vitro. Both transcripts were high in hNPCs, increased further on day 1, and started dropping subsequently to reach low levels in differentiated neurons. During astrocyte differentiation, they dropped sharply on day 1. BASP1-AS1 regulated the expression of BASP1, but not vice versa. BASP1-AS1 and BASP1 formed a molecular complex that also included the transcription factor TCF12. The TCF12 gene is coded by the DYX1 locus, and has been associated with inherited dyslexia and neurodevelopmental defects. Abrogation of either of the three components of the complex, impaired neuronal differentiation. Hence, the analysis of lncRNA biotypes in our study led to a divergent lncRNA-coding gene pair that was part of a novel neuronal differentiation pathway along with a transcription factor implicated in predisposition to inherited dyslexia.

## Results

### lncRNA biotypes during cortical neuronal differentiation

**Fig. 1:**
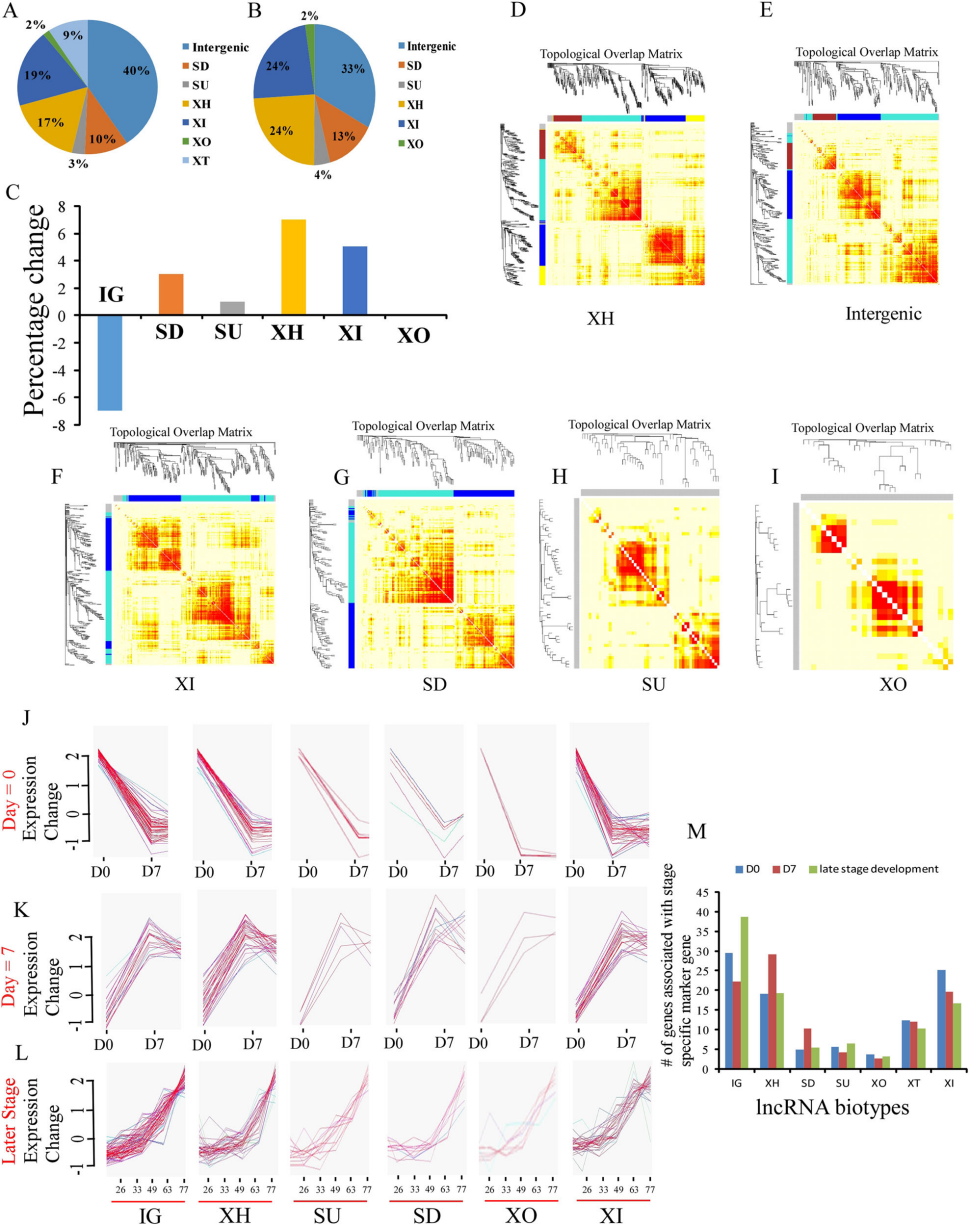
Weighted gene co-expression network analysis (WGCNA) and m-fuzz clustering analysis reveals the overall and stage-specific lncRNA biotypes during cortical neuronal differentiation. A synopsis of lncRNA biotypes during cortical neuronal differentiation. **a** Percentage of lncRNA biotype-associated coding genes with ≥1 RPKM value during in vitro cortecogenesis. **b** Percentage of lncRNA biotype-associated coding genes significantly involved in neuronal differentiation as per the Gene Ontology database. **c** Change in the percentage of lncRNA biotype-associated coding genes during neuronal differentiation as compared with overall cortical differentiation. **d**–**i** Topological overlapping matrix generated using WGCNA on expression data of different lncRNA biotypes. Clustering is based on the comparison of overall corticogenesis with neurogenesis. Maximum numbers of lncRNA gene-forming clusters during neuronal differentiation belong to the XH biotypes, followed by IG, XI, and SD. While SU and XO do not show any cluster with significantly higher membership score. **j**–**l** M-fuzz clustering analysis of different lncRNA biotypes at different stages of cortical neuronal differentiation. The upper panel shows the expression association of different biotypes of lncRNA-associated genes with D0- (Pluripotency) stage markers (POU5F (OCT4), NANOG, NODAL, and TDGF). The clustering sharply drops from day 0 to day 7. The middle panel shows the expression association of different biotypes of lncRNA genes associated with D7- (neuronal commitment) stage markers (PAX6 and SOX1). Clustering has sharply increased from day 0 to day 7. The lower panel shows the expression association of different biotypes of lncRNA-associated genes with later development-stage markers (EMX2, TBR1, CTIP2, CACNA1E, PRSS12, and CARTPT). **m** The number of lncRNA biotype-associated genes enriched at different stages of cortical neuronal development. IG with a maximum number of genes involved, which are around 30 and at D0 and later development stage, respectively, while at D7, XH biotypes showing the maximum involvement that is around 30.

We performed the comparative analysis of lncRNA biotypes^9,12,14^ during in vitro neuronal differentiation using publicly available data, following the biotype classification of Luo et al.^9^ (Supplementary Fig. S1). We analyzed the Cortecon data set (GSE56796)^13^, which provides RNA-seq data during in vitro cortical development (neuronal differentiation) from human embryonic stem cells (hESCs) for 77 days. About 73–95% of all coding genes were expressed at different stages of cortical development (Fig. 1a, b). Using Gene ontology, we estimated the percentages of different biotypes of lncRNA-associated coding genes involved in neuronal differentiation (as compared with overall cortical development). XH lncRNA-associated coding genes (7%) showed maximum enrichment with neuronal differentiation-related genes, which was followed by XI (5%), SD (3%), and SU (1%) biotypes. There was reduction in enrichment in IG and XO by 7%, and no change for XO (Fig. 1a–c).

### Clustering analyses to identify stage-specific lncRNA biotypes during corticogenesis

We constructed lncRNA biotype-specific co-expression networks using weighted gene co-expression network analysis (WGCNA) that combined all the temporal gene expression data of cortical development (day 0, day 7, day 12, day 19, day 26, day 49, day 63, and day 77). The combined data set of all the lncRNA genes during cortical development was clustered with the genes specifically altered during neuronal differentiation. Our analysis showed numerous transcriptional modules (Fig. 1d–i). XH lncRNA biotypes show maximum co-expression module (*n* = 4) that was followed by IG (*n* = 3), XI (*n* = 2), and SD (*n* = 2). SU and XO biotypes did not show any co-expression modules.

We used fuzzy c-mean clustering analysis to identify stage-specific clusters of biotypes during different days of neuronal differentiation during corticogenesis. Clusters were generated based on similar changes in expression patterns during corticogenesis and classified into different development stages as described in CORTECON^13^. We classified them into three categories: “pluripotency”, “neuronal commitment stage”, and “later development stage” based on the association (higher membership number) with genes from each category. The marker genes are Pluripotency—POU5F (OCT4), NANOG, NODAL, and TDGF corresponding to day 0; neuronal commitment stage—PAX6 and SOX1, corresponding to day 7; for later development stages (day 26 to day 77)—EMX2 (cortical specification), TBR1 (deep-layer formation), and CTIP2, CACNA1E, PRSS12, and CARTPT (upper-layer formation). IG and XI were higher at the pluripotency stage (D0) as compared with XH, while other biotypes remained very low. At day 7 or neuronal commitment stage, XH was most abundant (Fig. 1j–m).

**Fig. 2:**
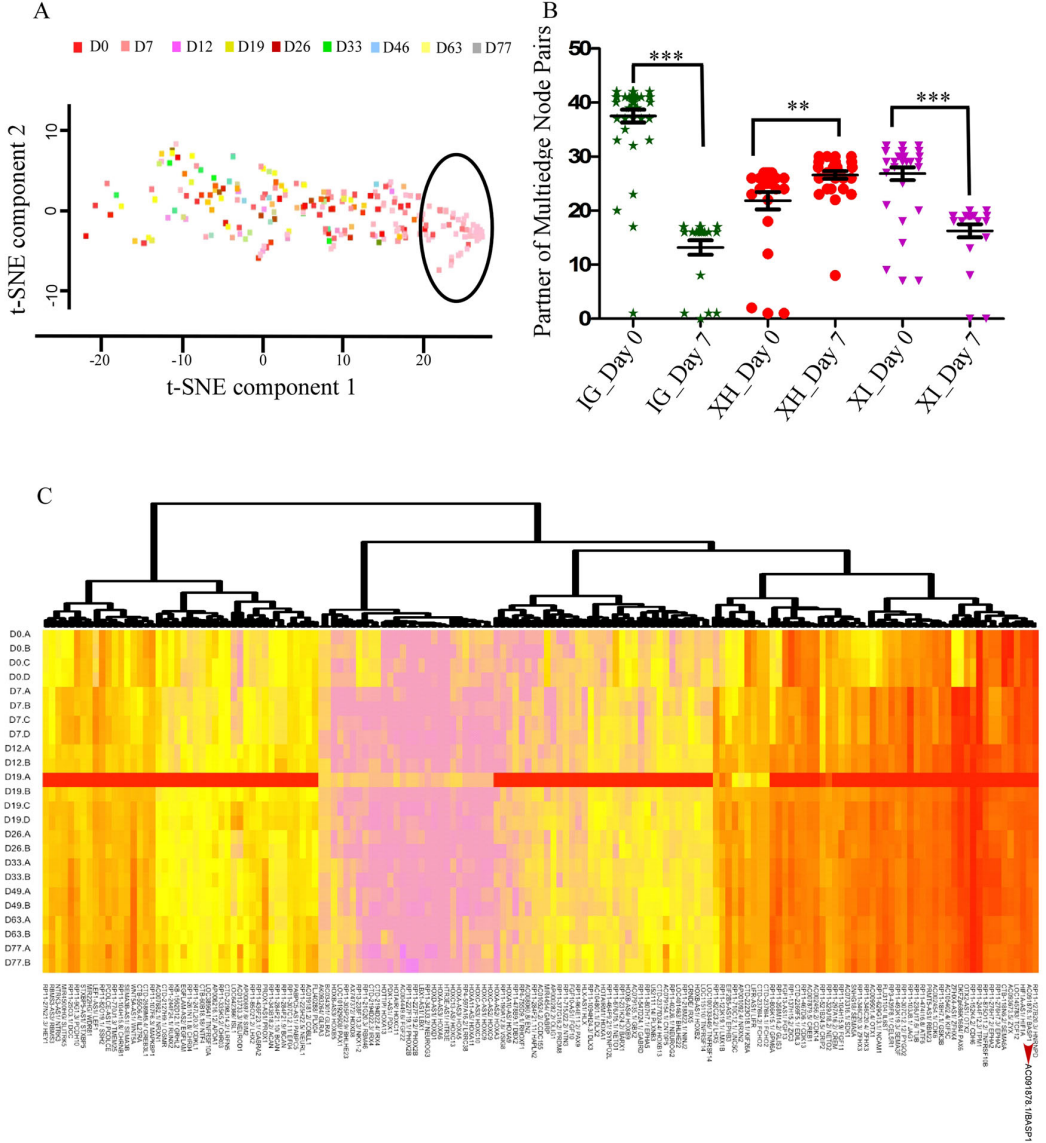
Dimension reduction (t-SNE) and partner of multiedge node pair analysis. **a** t-SNE analysis of XH biotype showing that the formation of the cluster is maximum at D7. None of the other biotypes showed any clustering by t-SNE at different days of differentiation (Supplementary Figs. S2, S3). Different colors represent genes at different days of cortical neuronal differentiation. **b** Estimation of a change in the network by multiedge node pair analysis of biotypes (IG, XH, and XI) from D0 to D7. **c** Hierarchal clustering of XH lncRNA biotypes during cortical neuronal differentiation. Heatmap showing expression of 160 XH lncRNA-associated genes during cortical development. Red arrow showing the position of BASP1 gene and its expression during all the stages of cortical neuronal differentiation.

To visualize the daywise cluster of these biotypes, we used t-distributed stochastic neighbor embedding (t-SNE) that computes principal components and cluster data on the basis of gene expression in two dimensions. Only the XH biotype formed a cluster on day 7. Other biotypes failed to form a cluster on any of the days (Fig. 2a; Supplementary Figs. S2a–c, S3a, b). Further, to understand the exact function of XH lncRNA biotype, we did GO analysis of only those lncRNA-associated genes that had a higher membership score with the D0 and D7 marker genes. The XH lncRNA-associated genes were highly enriched with the overall nervous system development on D0 (pluripotency stage) and on day 7, the neuronal commitment stage (Supplementary Figs. S4, S5).

Gene co-expression network analysis (for genes modulated in a similar manner) using Cytoscape, for the Cortecon data, from embryonic stem cells to neuronal commitment showed an increase in the network (measured by “partner of multiedge node pairs”) of the XH biotype from day 0 to day 7 (neuronal commitment stage) from 22 units to 26 units (*p* ≤ 0.005), while there was a drop in the network of other two biotypes from 38 units to 14 units and 26 units to 16 units for IG and XI, respectively (*p* ≤ 0.0005). These abundance, expression, and clustering analysis results show the prominent involvement of the XH biotype in cell fate determination and neuronal development (Fig. 2b).

### Identification of the BASP1 as the major hub gene during cortical neural differentiation

Among the XH-associated coding genes, we identified BASP1 as a coding partner of a key lncRNA (BASP1-AS1) associated with the neuronal commitment marker genes at D7 (Pax6 and Sox1). It was a HUB gene in association with other candidates using the Cytoscape Network Inference Toolbox (http://apps.cytoscape.org/) by ARACNe (Supplementary Fig. S6). It was also second in the list of genes identified by hierarchical clustering (Fig. 2c) for neuronal differentiation-associated genes. Hence, we studied the BASP1-AS1 lncRNA and its role in neuronal differentiation.

### BASP1-AS1 is localized in the nucleus and expressed in fetal brain

**Fig. 3:**
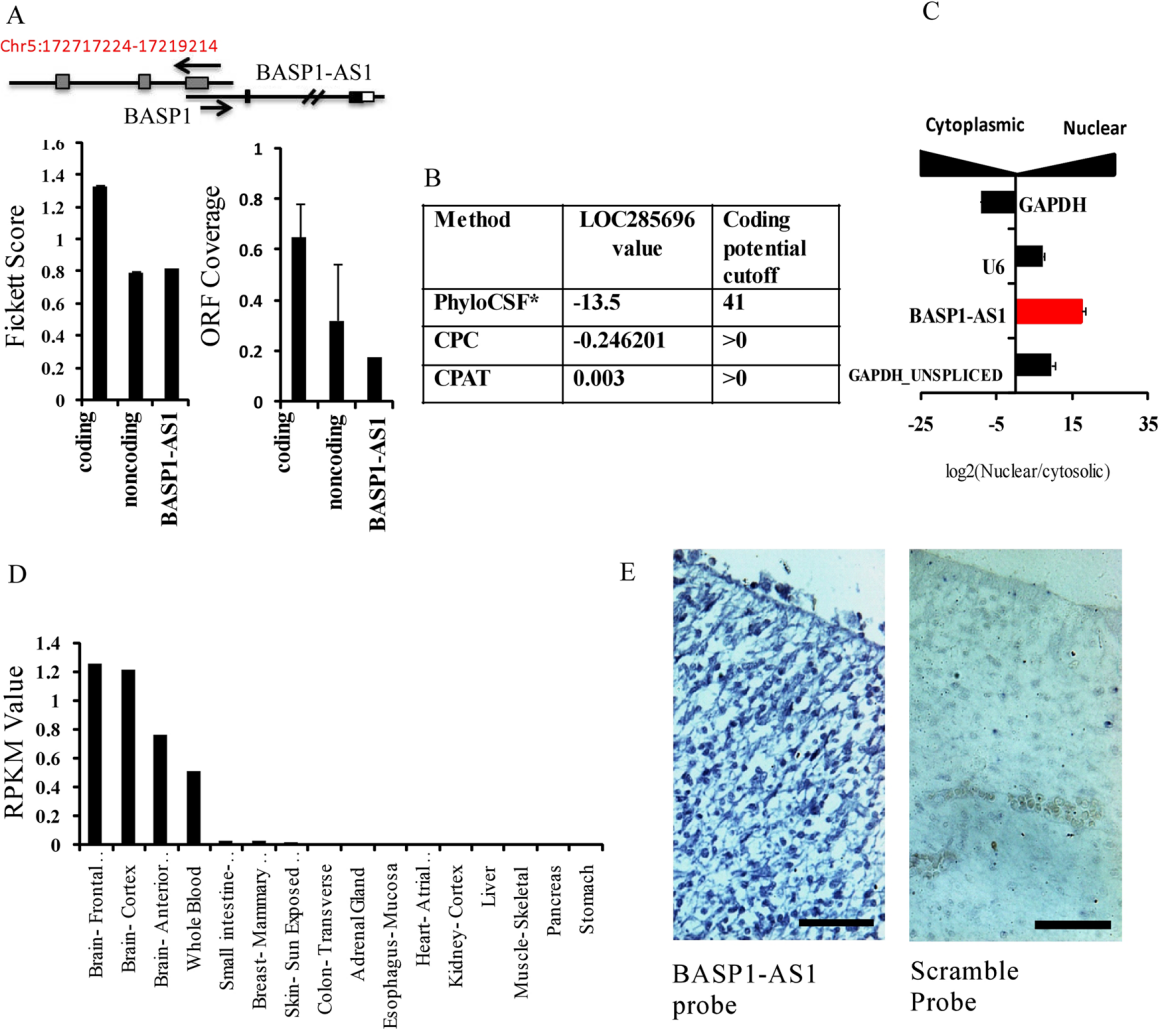
Identification and functional characterization of novel lncRNA loc285696 (*BASP1-AS1*). **a** Schematic diagram of the BASP1-AS1 lncRNA and its coding partner BASP1. Fickett score and ORF coverage of 50 coding and noncoding transcripts, and comparison of these scores with the *BASP1-AS1* transcript, indicates that it is a noncoding transcript. **b** Table showing BASP1-AS1 coding potential values and its cutoff using different coding potential calculators. For PhyloCSF, a score lower than 41 represents that the transcript is noncoding; a score above the cutoff is likely to be coding. PhyloCSF value was taken from LNCpedia with ID: lnc-*BASP1*–*9*, location (hg19): chr5:17202383-17203254. In the same manner for CPC, a score equal to or lower than zero cutoff means that the transcript is noncoding. Same is the cutoff for CPAT, where a score near zero indicates that it is noncoding. By all the three indications, BASP1-AS1 is a noncoding transcript. **c** Nuclear and cytosolic fractionation followed by RT-qPCR for indicated mRNAs and for *BASP1-AS1* (represented as log2 expression values). The result indicates nuclear localization of the *BASP1-AS1* as a feature of lncRNAs. As expected, control GAPDH was cytoplasmic, while unspliced GAPDH and U6 were predominantly nuclear (*n* = 3, mean ± SD of three independent experiments). **d** Localization of *BASP1-AS1*. Reads per kilobase per million (RPKM) values for BASP1-AS1 transcript (AC091878.1) in indicated tissue samples, as obtained from the GTEx RNA-seq database indicated that it was mainly expressed in the CNS and whole blood. **e** ISH for *BASP1-AS1* in human SVZ fetal brain sections, indicating its expression in human fetal brain.

The UCSC genome browser shows that the lncRNA LOC285696 located at 5p (UCSC database transcript ID AC091878.1 and GENCODE database ID ENST00000399760.2, hitherto referred as *BASP1-AS1*) is an uncharacterized divergent RNA (XH biotype), transcribed in the opposite direction to the protein-coding gene BASP1 (Fig. 3a). The 5′ ends of the lncRNA and *BASP1* gene share an overlapping region of 599 bps. *BASP1-AS1* lncRNA is 3316-bp long with three exons.

The low score of *BASP1-AS1* (Fig. 3a) by the Fickett score^15^ and ORF coverage^16^ and phyloCSF^17^, CPC^18^, and CPAT tool^19^ analysis indicated that *BASP1-AS1* has no coding potential (Fig. 3b). RT-qPCR in nuclear and cytoplasmic fractions of hNPCs showed that BASP1 transcript was over 15-fold greater in the nucleus than the cytoplasm, a feature of noncoding RNAs (Fig. 3c). *BASP1-AS1* is expressed largely in different regions of the brain, in addition to whole blood in the genotype-tissue expression project (GTEx) RNA-seq^20^ database (Fig. 3d). In situ hybridization in human fetal brain sections showed also demonstrated its abundant expression (Fig. 3e). GTEx data sets showed the highest expression of both BASP1 and BASP1-AS1 in the frontal cortex (Supplementary Fig. S7a, b). Although the expression of BASP1 was higher than BASP1-AS1, they were highly correlated (*R*^2^ = 0.97) in all the brain regions (Supplementary Fig. S7c). Based on databases and experimental results, one can say that BASP1-AS1 is a noncoding lncRNA expressed in the brain, including the developing brain, in a manner very similar to its coding gene pair BASP1.

**Fig. 4:**
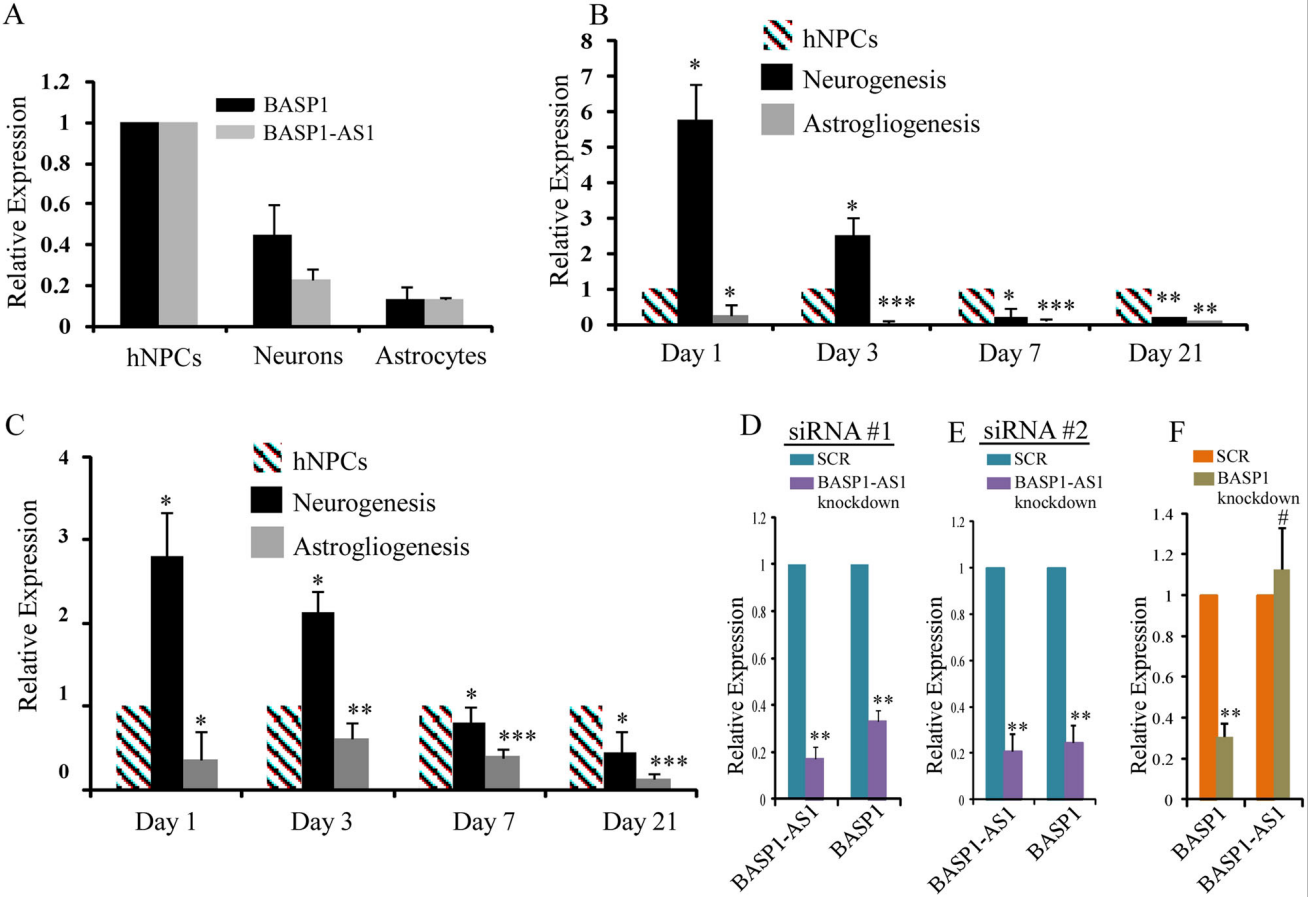
Expression of *BASP1-AS1* and *BASP1* during hNPC differentiation. **a** Relative expression of both BASP1-AS1 and BASP1 in NPCs (maximum) followed by mature neurons and astrocytes (*n* = 3 biological replicates, mean ± SD). It is the highest in hNPCs, less in neurons, and least in astrocytes. **b**, **c** They show the relative expression of BASP1-AS1 and BASP1, respectively during different days of neural and astroglial differentiation from hNPCs. Both the transcripts show a marked rise on day 1 followed by a subsequent fall during neural differentiation. However, during astroglial differentiation, there was a sharp fall on day 1. *p ≤ 0.05, **p ≤ 0.005, and ***p ≤ 0.0005 (*n* = 3 biological replicates, mean ± SD, Student's two-tailed *t* test). **d**, **e** This figure shows that BASP-AS1 knockdown results in a fall in BASP1 transcript levels in hNPCs. **p ≤ 0.005 (*n* = 3 biological replicates, mean ± SD, Student's two-tailed *t* test). **f** This figure shows that BASP-AS1 knockdown results in a fall in BASP1 transcript levels in hNPCs. **p ≤ 0.005 (*n* = 3 biological replicates, mean ± SD, Student's two-tailed *t* test), but no significant effect on BASP1-AS1 transcript on BASP1 knockdown.

### *BASP1-AS1* regulates expression of BASP1 during the neuronal differentiation of fetal-derived hNPCs

We have studied the expression of BASP1 and BASP1-AS1 transcripts during lineage-specific differentiation of hNPCs, following the pre-established protocol for hNPC maintenance and differentiation^21–23^ (Supplementary Fig. S8a–f). We observed that the expression of both *BASP1-AS1* and *BASP1* paralleled each other, which was the highest in the undifferentiated hNPCs, significantly lower in neurons, and least in astrocytes (Fig. 4a). When the hNPCs were differentiated in vitro, the expression of both the transcripts was moderately high in hNPCs, rose sharply on the first day of neuronal differentiation (BASP1-AS1 = 5.7-fold, *p* ≤ 0.05, and BASP1 = 2.8-fold, *p* ≤ 0.05), and then fell markedly by day 3 and continued at a much lower level. Astrocytic differentiation resulted in marked reduction (BASP1-AS1 = 75% reduction, *p* ≤ 0.05, and BASP1 = 64% reduction, *p* ≤ 0.05), on day 1 itself (Fig. 4b–c). The experiment was continued till day 21.

**Fig. 5:**
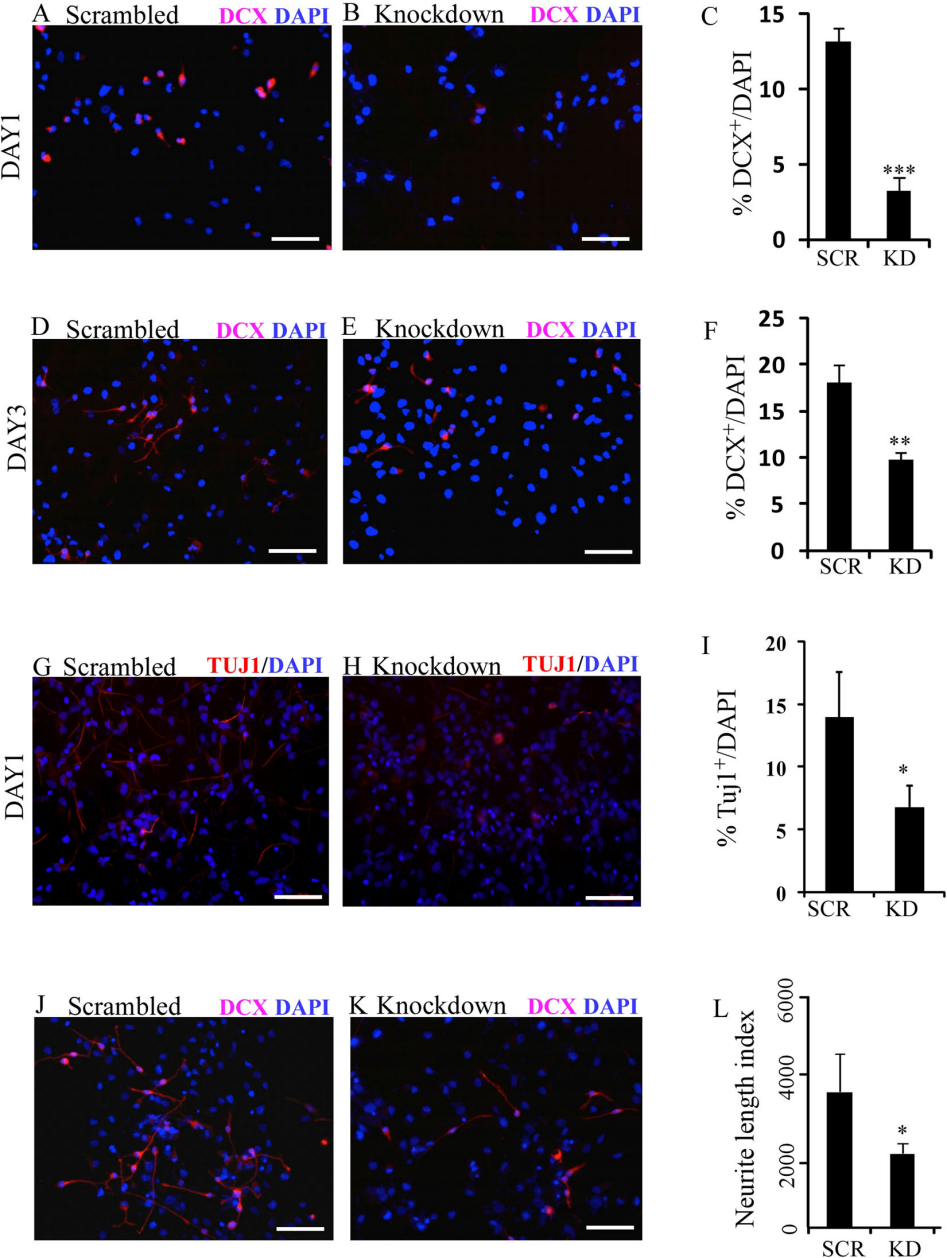
BASP1-AS1 affects neuronal differentiation and neurite length of hNPCs. **a**–**f** Percentage of DCX + cells decreases significantly after BASP-AS1-KD as compared with scrambled controls on both DAY1 and DAY3 of neural differentiation, respectively. We found that there were approximately fourfold higher DCX + cells in control as compared with BASP1-AS1-KD samples on day 1 of neural differentiation, and approximately twofold higher on day 3. **p ≤ 0.005 and ***p ≤ 0.0005. Scale bar, 50 μm (*n* = 3 biological replicates, mean ± SD, Student's two-tailed *t* test). **g**–**i** Percentage of Tuj1^+^ cells decreases significantly after BASP-AS1-KD as compared with scrambled controls on DAY1 of neuronal differentiation. We found that there were approximately twofold higher Tuj1^+^ cells in control as compared with BASP1-AS1-KD samples on day 1 of neuronal differentiation. *p ≤ 0.05. Scale bar, 50 μm. (*n* = 3 biological replicates, mean ± SD, Student's two-tailed *t* test). **j**–**l** There is a significant decrease in neurite length on DAY3 of neural differentiation in BASP1-AS1-KD as compared with scrambled controls. As determined by ImageJ with plugin called “Neurite Tracer”. *p ≤ 0.05. Scale bar, 50 μm (*n* = 3 biological replicates, mean ± SD, Student's two-tailed *t* test).

Knockdown of *BASP1-AS1* in the hNPCs markedly reduced the *BASP1* transcript (66% reduction, *p* ≤ 0.005) (Fig. 4b–c), though knockdown of the *BASP1* gene in hNPCs had no effect on *BASP1-AS1* expression, indicating that *BASP1-AS1*, like other divergent lncRNAs, regulates *BASP1* expression (Fig. 4d–f)^9,24^.

### BASP1-AS1 influences neuronal differentiation

We conducted knockdown experiments on days 1 and 3 of neuronal differentiation of hNPCs. There was a significant reduction in the number of cells staining for the early neural differentiation marker, double cortin (DCX) both on days 1 and 3, and Tuj1 on day 1 as seen by immunohistochemistry, indicating impaired neuronal differentiation. The percentage of DCX- positive cells was reduced from 13.1% ± 0.94% to 3.2% ± 0.84% (*p* ≤ 0.0005) on day 1, and 18.01% ± 2.02% to 9.8% ± 0.80% (*p* ≤ 0.005) on day 3 (Fig. 5a–f). The percentage of Tuj1-positive cells was reduced from 13.9% ± 3.6% to 6.7% ± 1.70% on day 1 (Fig. 5g–i) (*p* ≤ 0.05). The live/dead assay showed no significant difference between scrambled and BASP1-AS1 siRNAs (Supplementary Fig. S9a–f).

As BASP1 has been shown to induce neurite outgrowth^25^, we studied the effect of BASP1-AS1 knockdown on neurite formation. On day 3 of neural differentiation, there was a reduction of 1.8-fold in the neurite length (from 3546.79 ± 977.5 units to 1934.63 ± 249.37) (Fig. 5j–l). These experiments strongly implicate the role of *BASP1-AS1* in neuronal differentiation.

**Fig. 6:**
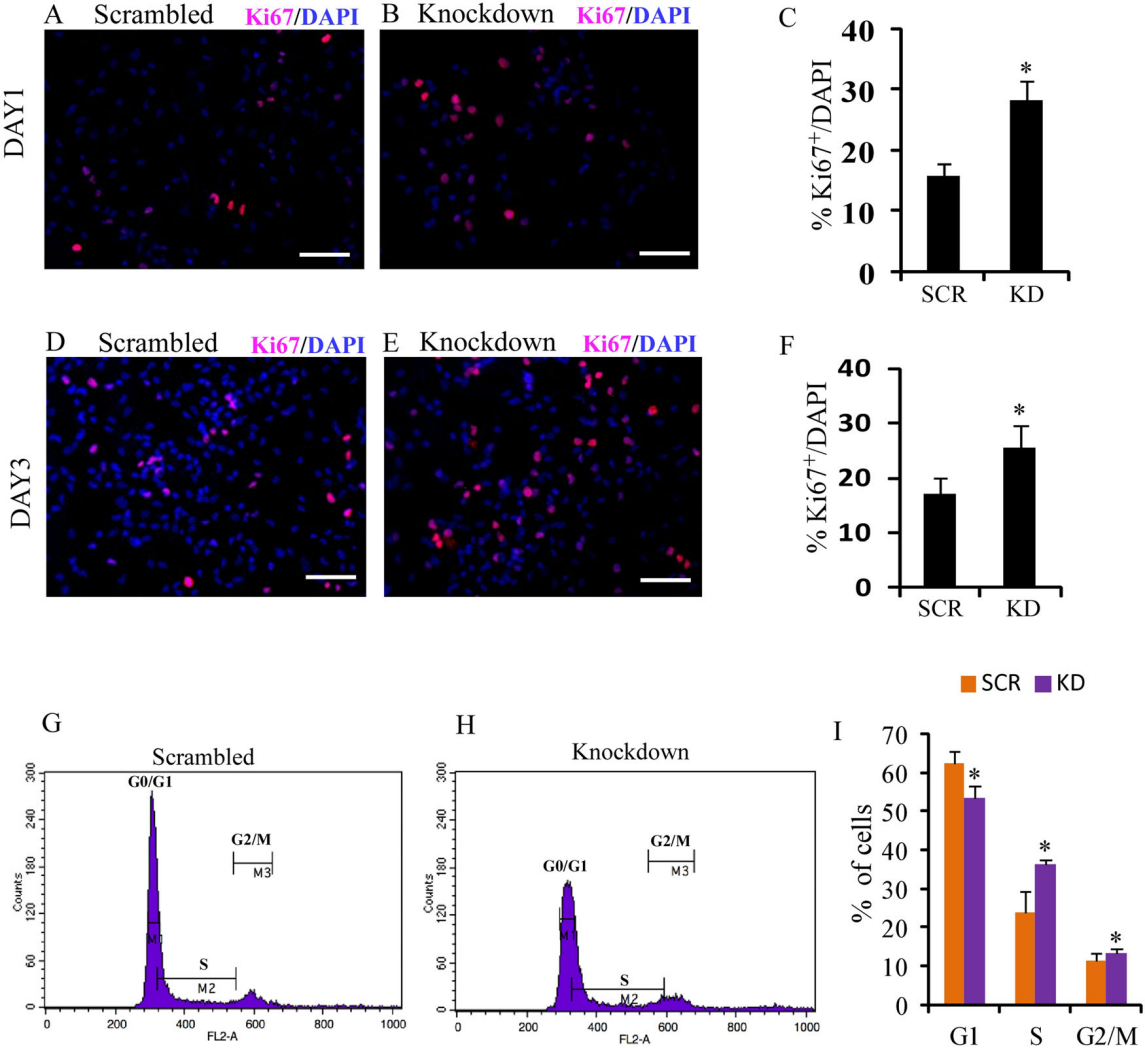
BASP1-AS1 affects neuronal proliferation and alters cell-cycle dynamics. **a**–**f** There is an increase in Ki67-positive cells significantly after BASP1-AS1-KD as compared with scrambled controls on both DAY1 and DAY3 of neural differentiation, respectively. *p ≤ 0.05. Scale bar, 50 μm (*n* = 3 biological replicates, mean ± SD, Student's two-tailed *t* test). **g**, **h** Representative images of DNA content analysis by propidium iodide (PI) staining using FACS 24 h post transfection of either scrambled siRNA or BASP1-AS1 siRNA in human hNPCs. **i** Bar graph shows quantitative analysis of data obtained from PI staining depicting the percentage of cells in G0/G1, S phase, and G2/M phase of the cell cycle in indicated groups. *p ≤ 0.05 (*n* = 3 biological replicates, mean ± SD, Student's two-tailed *t* test).

BASP1-AS1 knockdown was also associated with higher cell proliferation, as indicated by increased Ki67-positive cells, from 15.7% ± 2.12% in the hNPC culture to 28.17% ± 3.3% on day 1 after knockdown (*p* ≤ 0.05) (Fig. 6a–c). On day 3, the increase was from 17.1% ± 2.74% to 25.5% ± 4.11% (*p* ≤ 0.05) (Fig. 6d–f).

FACS analysis showed an increase in the percentage of cells in the S phase in hNPCs 24 h post knockdown. The percentage of cells in the G0/G1, S, and G2/M phase of the cell cycle in knockdown samples was 53.53 ± 3.10%, 36.12 ± 1.4%, and 13.4 ± 1.10% and 62.53 ± 2.76% (*p* ≤ 0.05), 23.76 ± 5.36% (*p* ≤ 0.05), and 11.34 ± 1.85% (*p* ≤ 0.05), in the scrambled, respectively (Fig. 6g–i).

### Molecular interaction involving *BASP1-AS1* and the *BASP1* genomic locus

**Fig. 7:**
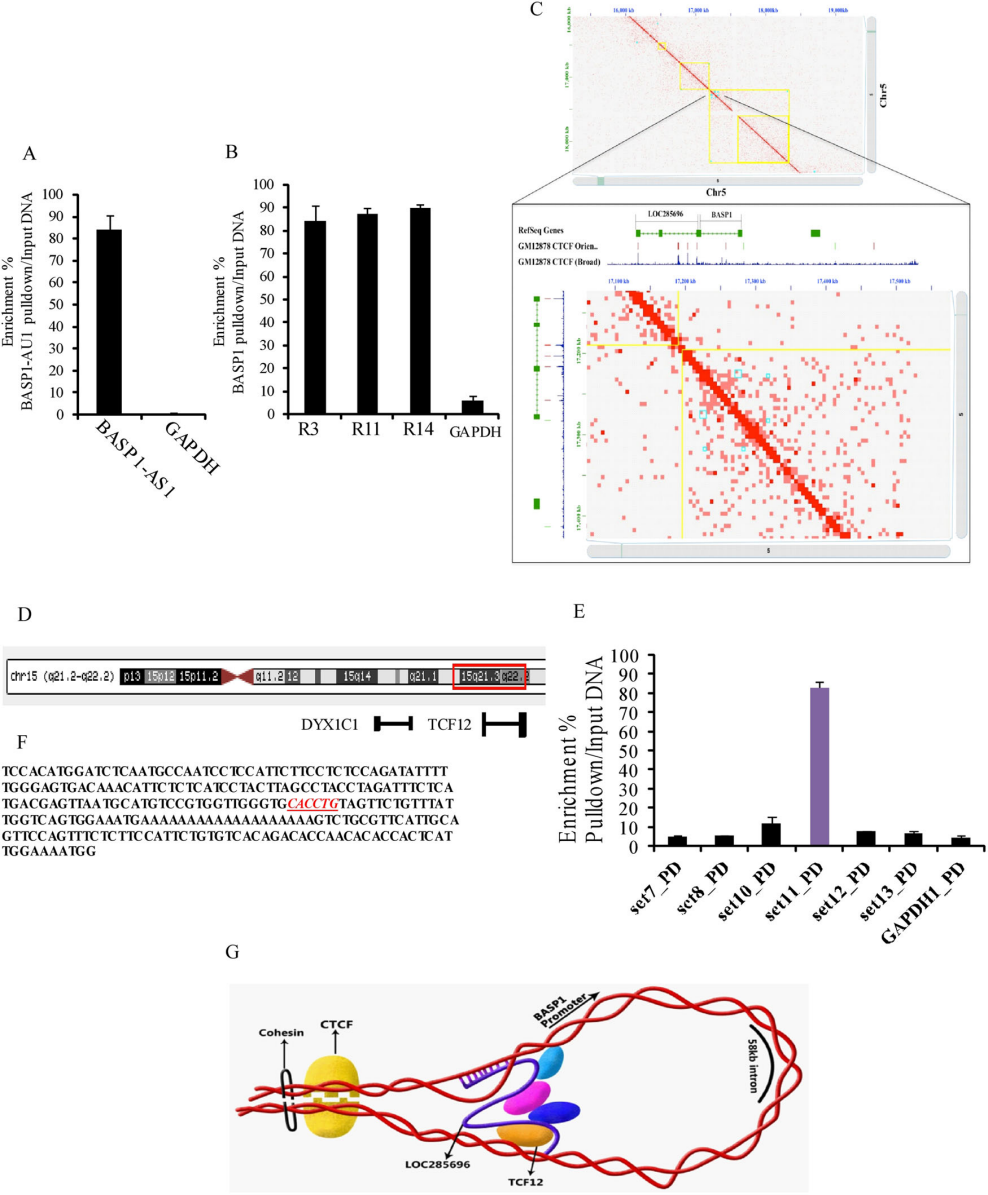
Molecular interaction involving *BASP1-AS1, BASP1* genomic locus, and transcription factor TCF12. **a** Bar graph showing *BASP1-AS1* retrieval after ChIRP followed by qPCR showed that ChIRP retrieved about 80% of *BASP1-AS1* RNA, while GAPDH was almost undetectable. **b** Pulldown of different genomic regions of BASP1. Regions of the *BASP1* gene corresponding to R3, R11, and R14 were enriched maximally. R3 that is located at the 5′ end and R11 and R14 at the 3′ end of the 58-kb BASP1 gene showed the maximum pulldown. R1, R2, R4–R10, and R12 and R13 had undetectable levels of the target (*n* = 3 biological replicates, mean ± SD). **c** Hi-C data of GM12878 represent loop formation at the genomic locus of *BASP1*. This image was generated using Juicebox package. The GM12878 cells Hi-C data set was used to visualize the contact domain within the *BASP1* gene locus. The matrix with a resolution of 0.5 kb is shown, indicating genomic locations of *BASP1* and LOC285696 (*BASP1-AS1*—chr5: 172717224-17219214), CTCF sequence orientation (red represents “reverse”—chr5: 17216900 and green represents “forward” orientation—chr5: 17283500), and CTCF-binding track. The presence of a chromatin loop is indicated in the track with annotated peaks (cyan, peak1—chr5: 17220001-17230000 and peak2—chr5: 17270001-17280000). Chromatin loops are frequently anchored with the convergent CTCF motifs; here at *BASP1* genomic locus, both the CTCF sequences read in a convergent fashion as shown in panels 2 and 3. **d** Image adopted from UCSC browser showing proximity of *DYX1C1* and *TCF12*, both genes being components of the *DYX1* locus. **e** Enrichment of the R11 region of the *BASP1* gene after ChIP analysis following *TCF12* pulldown, indicating binding of TCF12 to the specific region (*n* = 3 biological replicates, mean ± SD). **f** The E-box-binding domain on the R11 fragment (red) that has been earlier predicted to bind to *TCF12*. **g** A regulatory model showing the interaction of *BASP1-AS1* RNA, *BASP1*, and *TCF12* and possible other transcription factors, to form a looped structure.

Divergent lncRNAs regulate the corresponding genes by direct interactions with the coding gene^9^. To elucidate the interaction of *BASP1* gene and *BASP1-AS1* RNA, we performed chromatin isolation by RNA purification (ChIRP)^26^. In ChIRP, tiling biotinylated oligonucleotides were designed specific to BASP1-AS1 to retrieve the lncRNA-bound DNA, measured by qPCR (Supplementary Fig. S10a–b). We designed 14 primer pairs mapping to exon 1, exon 2, and to 1500 bp upstream and 1000 bp downstream of the BASP1 gene (Supplementary Fig. S10c). *BASP1-AS1* RNA pulldown was done for each biotinylated probe using streptavidin-coated magnetic beads, followed by real-time PCR to identify the enriched genomic regions of *BASP1*. BASP1 has two exons separated by ~58-kbp intron^27^. The *BASP1-AS1* gene is about 3.3 kb, and is transcribed in the opposite orientation. Both overlap by 599 bps in the 5′ region. Of the 14 segments (R1–14), spanning the entire 5′ and 3′UTR and the exons of the *BASP1* gene we studied, only three regions—R3 (5′UTR), R11 (spanning exon 2 and adjacent 3′UTR,) and R14 (3′UTR) were enriched (Fig. 7a, b). The locations of interacting regions at the two ends of the long (~58 kb) *BASP1* gene suggest the formation of a looped structure.

Genomic looping has been demonstrated in literature by Hi-C technique. We have used Hi-C database of GM1287, the B-lymphocyte origin cells generated earlier^28^, to identify genomic looping in the BASP1 locus^29^. We identified a looped structure at *BASP1* gene ends in GM12878 cell lines. Also, chromatin loops are frequently anchored with the convergent CTCF motifs. The matrix with a resolution of 0.5 kb is shown, indicating genomic locations of *BASP1* and LOC285696 (*BASP1-AS1*—chr5: 172717224-17219214) and their CTCF sequence orientation (Fig. 7c). The presence of both CTCF sequences and a chromatin loop signature is indicative of loop formation at the *BASP1* locus.

### Interaction of the transcription factor TCF12 with the BASP1 gene

The UCSC ChIP-seq database showed the binding of TCF12, among the different transcription factors. TCF12 is a product of the *DYX1* locus, linked to dyslexia^30^ (Supplementary Fig. S2). The DYX1 locus comprises two genes DYX1C1 and TCF12 (Fig. 7d). TCF12 is linked to expansion of neural stem cells (NSCs) and NPCs during neurogenesis^31^. It is also reported to be associated with early cell fate determination of progenitors into neurons in the midbrain^32^. CHIP assay using the *TCF12* antibody (Fig. 7e) demonstrated a strong signal at R11 at the 3′ end of the *BASP1* gene (region R11). R11 was also positive in the ChIRP assay for the *BASP1-AS1*/*BASP1* gene interaction. The TCF12 is a member of the basic helix–loop–helix (bHLH) E-protein family that recognizes the consensus-binding site (E-box) CANNTG^33,34^. The sequence of R11 (on BASP1) pulldown has this E-box motif (Fig. 7f). A pictorial representation based on the above findings depicts the possible molecular complex (Fig. 7g). However, the role of other putative transcription factors in this regulation has yet to be established.

### All three components of the complex, *BASP1-AS1*, *BASP1*, and *TCF12*, are essential for neural differentiation from hNPCs

**Fig. 8:**
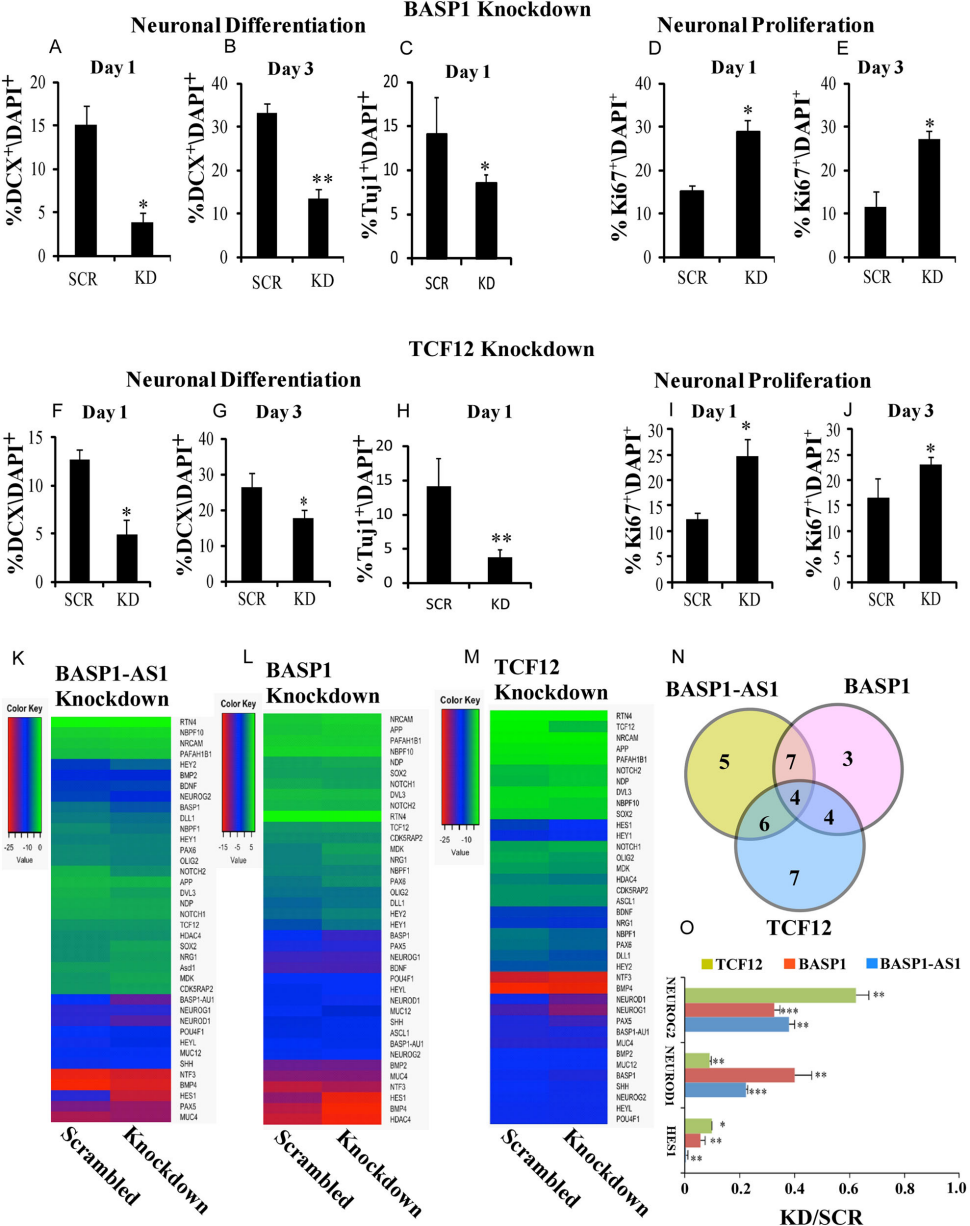
*BASP1-AS1, BASP1*, and *TCF12* are essential for neural differentiation from hNPCs. **a**, **b** Percentage of DCX + cells decreases significantly after BASP1-KD as compared with scrambled controls on both DAY1 and DAY3 of neural differentiation, respectively. We found that there were approximately threefold higher DCX + cells in control as compared with BASP1-KD samples on day 1 of neural differentiation and approximately twofold higher on day 3. **p ≤ 0.005 and *p ≤ 0.05 (*n* = 3 biological replicates, mean ± SD, Student's two-tailed *t* test). **c** Percentage of Tuj1^+^ cells decreases significantly after BASP1-KD as compared with scrambled controls on DAY1 of neuronal differentiation. We found that there were approximately twofold higher Tuj1^+^ cells in control as compared with BASP1-KD samples on day 1 of neuronal differentiation. *p ≤ 0.05. (*n* = 3 biological replicates, mean ± SD, Student's two-tailed *t* test). **d**, **e** There is significant increase in Ki67-positive cells after BASP1-KD as compared with scrambled controls on both DAY1 and DAY3 of neural differentiation, respectively. *p ≤ 0.05 (*n* = 3 biological replicates, mean ± SD, Student's two-tailed *t* test). **f**, **g** Percentage of DCX + cells decreases significantly after TCF12-KD as compared with scrambled controls on both DAY1 and DAY3 of neural differentiation, respectively. *p ≤ 0.05 (*n* = 3 biological replicates, mean ± SD, Student's two-tailed *t* test). **h** Percentage of Tuj1^+^ cells decreases significantly after TCF12-KD as compared with scrambled controls on DAY1 of neuronal differentiation. **p ≤ 0.005 (*n* = 3 biological replicates, mean ± SD, Student's two-tailed *t* test). **i**, **j** There is significant increase in Ki67-positive cells after TCF12-KD as compared with scrambled controls on both DAY1 and DAY3 of neural differentiation, respectively. *p ≤ 0.05 (*n* = 3 biological replicates, mean ± SD, Student's two-tailed *t* test). **k, m** Heatmaps showing the effect of *BASP1-AS1*, *BASP1*, and *TCF12* knockdown, respectively on an array of neurogenesis-related genes. **n** Venn diagram illustrating the differential expression of overlapping genes in three indicated knockdown conditions. **o** Bar graph showing that a common set of three genes (NEUROG2, NEUROD1, and HES1) were significantly downregulated in all the indicated three conditions of knockdown (BASP1-AS1, BASP1, and TCF12). *p ≤ 0.05, **p ≤ 0.005. The results are expressed in the ratio of knockdown/scrambled (KD/SCR) (*n* = 3 biological replicates, mean ± SD, Student's two-tailed *t* test).

To study the impact of BASP1 and TCF12 on neurogenesis, we conducted knockdown experiments on days 1 and 3 of neuronal differentiation of hNPCs. Knockdown of *BASP1* in hNPCs resulted in impaired neural differentiation as indicated by a significant reduction in the number of cells staining for the early neural differentiation marker, double cortin (DCX) on days 1 and 3: from 15.01% ± 2.2% to 3.9% ± 1.04% (*p* ≤ 0.05) on day 1 and 33.04% ± 2.21% to 13.5% ± 2.12% (*p* ≤ 0.005) on day 3 (Fig. 8a, b). On day 1, Tuj1-positive cells reduced from 14.15% ± 4.06% to 8.63% ± 0.8% (*p* ≤ 0.05) (Fig. 8c). There was also higher cell proliferation, as indicated by increased Ki67-positive cells (Fig. 8d–e), which increased from 15.35% ± 1.03% to 29.02% ± 2.64% on day 1 and from 11.6% ± 3.4% to 27.2% ± 1.8% on day 3 (*p* ≤ 0.05).

For TCF12 knockdown, the percentage of DCX-positive cells reduced from 12.67% ± 1.07% to 4.9% ± 1.55% (*p* ≤ 0.005) on day 1 and from 27.23% ± 4.21% to 15.43% ± 2.26% on day 3 (*p* ≤ 0.05) (Fig. 8f–g). Tuj1-positive cells reduced from 14.15% ± 4.06% to 3.80% ± 1.08% (*p* ≤ 0.005) (Fig. 8h) on day 1. This was also associated with increased Ki67-positive cells (Figure 8i–j), which increased from 12.49% ± 1.20% to 24.68% ± 3.32% (*p* ≤ 0.05) on day 1 and from 16.63% ± 3.71% to 23.2% ± 1.33% (*p* ≤ 0.05).

A panel of 37 genes involved in hNPC differentiation was studied using quantitative RT-PCR after knockdown of *BASP1-AS1*, *BASP1*, or *TCF12* of proliferating hNPCs. Gene expression was measured on the first day of differentiation (8k–m). Three genes, *NEUROD1, NEUROG2*, and *HES1*, which are important in neural differentiation and in NOTCH signaling, were downregulated by all (Fig. 8n–o). While the panel is by no means exhaustive, the results indicate a commonality of the downstream process of the three components of the complex, and further support the involvement of all three components in neurogenesis.

## Discussion

The annotated number of lncRNA genes has surpassed the number of coding genes in the genome, highlighting their important roles in biological processes, including cellular differentiation and cell fate determination. These include coding gene-associated lncRNAs that control the expression of their neighboring coding genes. Most reports point out to the important role of the divergent lncRNA biotype in cell development. In our previous study, we observed that divergent lncRNAs are more involved during neuronal, but not in astrocytic differentiation^23^. Here, we initially studied the comparative role of different lncRNA biotypes during cortical neuronal differentiation. Our analyses suggest that for cortical neural differentiation, three biotypes XH, IG, and XI could be involved. Based on different forms of clustering and network analyses, we felt that the XH biotype was more prominent in cortical neuronal differentiation.

Hub gene identification by ARACNe of coding genes associated with XH lncRNAs showed that the BASP1 forms the major hub gene during neuronal differentiation. It was also second in the list of genes identified by hierarchal clustering. We investigated the divergent lncRNA associated with BASP1 (BASP1-AS1) to further understand its role during neuronal differentiation. Even though this lncRNA has been reported in the databases as loc285696 (UCSC), its functions are as yet unknown. *BASP1*, a suppressor of Wnt signaling, is a nerve-ending “signal” protein. It is highly expressed in neurons during brain development^25^, and induces neuronal differentiation in K562 cells^35^ and neurite growth in hippocampal neurons^25^. We have been able to demonstrate that BASP1-AS1 regulates BASP1 in hNPCs, and has a critical role in neuronal differentiation.

These form two components of a molecular complex that we have described. The third component of the complex is *TCF12*, a protein coded by the DYX1 locus replicated in several studies in inherited dyslexia. There have also been suggestions of the role of *TCF12* in neurodevelopment because of its implication in craniostenosis^36^ and increased transcription during expansion of precursor cell population during rodent neurogenesis^31^. Our study has identified a distinct role for the same in progenitor differentiation. Though an understanding of the exact mechanism by which the complex induces neuronal differentiation is still preliminary, this study has identified a set of common genes that are regulated by the complex. It appears that both the lncRNA and TCF12 finally influence the expression of BASP1, which then participates in subsequent differentiation steps.

To conclude, our study has not only identified a novel role for a lncRNA in the neuronal differentiation of hNPCs, but also shown the critical contribution of the complex comprising the divergent lncRNA, coding gene, and a transcription factor in the process. While there have been strong indicators of the role of both BASP1 and TCF12 in neurogenesis, we have been able to demonstrate their specific functions in the process of neuronal differentiation of hNPCs, and identify a novel pathway critical to this process. We have also been able to indicate a link connecting inherited dyslexia to neuronal differentiation through TCF12. Our work also indicates the importance of identifying divergent lncRNAs involved in specific developmental processes to obtain leads that could be used to identify their cellular and molecular regulatory roles.

## Materials and methods

### Bioinformatics analysis

#### M-fuzz clustering for stage-specific cluster identification during neurogenesis

For identification of stage-specific lncRNA biotype cluster from the different days of differentiation of RNA-seq corticogenesis data set, we have performed the nonhierarchical fuzzy c-means (FCM) clustering algorithm using M-fuzz and Mfuzzgui package^37^ in the Bioconductor R-based package (R Development Core Team 2011) available at https://www.biologie.hu-berlin.de/en/gruppenseiten-en/sfb618/publications/Kumar2007. M-fuzz clustering is a soft clustering, which produces gradual membership values of a gene between 0 and 1 representing the membership of this gene for a particular tightly co-expressed gene cluster. Fuzzy c-means produces gradual membership values µ_ij_ of a gene i between 0 and 1, indicating the degree of membership of this gene for cluster j. At this point, the standardization method was time based, the Fuzzy C-means (m) taken as 1.25 and minimum membership value (min.acore) as 0.5. Thus, soft clustering can effectively reflect the strength of a gene’s association with a cluster. Gradual membership values allow the definition of cluster cores of tightly co-expressed genes.

#### t-Distributed stochastic neighbor embedding (t-SNE)

For visualization of stage-specific biotype cluster, we have used t-SNE and Rtsne packages for R environment (R Development Core Team 2011). t-Distributed stochastic neighbor embedding (t-SNE) is a nonlinear dimensionality reduction algorithm predominantly well appropriate for the exploring of high-dimensional data sets. t-SNE reduced the discrepancy between two distributions, and discerned patterns in data by identifying an observed cluster based on the resemblance of data points with various features. t-SNE and Rtsne are a robust way to spot daywise specific cluster of a particular biotype during neurogenesis.

#### Weighted network analysis of Cortecon data sets

In order to perform the overall biotype-wise correlation network analysis of transcriptomic data of neurogenesis (Cortecon data set after GO enrichment analysis), we first tied the lncRNA biotype files having genes enriched with neuronal differentiation process using Galaxy tool (https://usegalaxy.org/) and then filtered out biotype (IG, XH, XI, SD, SU, and XO)-associated gene involved during neurogenesis. Based on stage-specific markers, day 0 was taken as equivalent to ESCs (ESC makers are OCT4, NANOG, NODAL, and TDGF), day 7 as an equivalent of human neural progenitor cells (hNPCs) (hNPC markers—PAX6 and SOX1). Subsequent stages indicated differentiated neurons (markers—EMX2, TBR1, CTIP2, CACNA1E, PRSS12, and CARTPT).

We have used WGCNA package^38^ from Comprehensive R Archive Network (CRAN) and dependencies from Bioconductor. To uncover modules of highly correlated genes, we started with generation of Pearson correlations and then transformed into an unsigned adjacency matrix by means of a power function. This produce scale-free adjacency matrix, i.e., the weighted co- expression network. Further, the adjacency matrix was converted into a topological overlap matrix (TOM); we used hierarchical clustering to group genes derived from the topological overlap of their connectivity to spot a cluster of highly co-regulated genes; each module was designated a distinctive (and arbitrary) color identifier.

#### Key network identification within stage-specific biotype cluster

After identification of Stage specific biotype cluster via fuzzy means algorithm, we performed network analysis through Network Analyzer tool in Cytoscape^39,40^ (https://cytoscape.org/). We used ARACNE (Algorithm for the Reconstruction of Accurate Cellular Networks)^41^ algorithm for the identification of key network and hub gene within a stage-specific cluster available through the Cyni Toolbox^42^ panel under the Infer Network plugin in Cytoscape.

### Cell culture

Handling of human tissues was carried out by the guidelines of Institutional Human Ethics Committee and Stem Cell and Research Committee of National Brain Research Centre, India, and Indian Council of Medical Research (ICMR), India. A well-characterized culture system of human neural precursor cells (hNPCs) derived from telencephalon of 10–15-week-old aborted human fetuses was employed for the study, as described previously^21^. Derived hNPCs were >99% positive for Nestin (neural stem cell marker) and formed neurospheres with a high degree of efficiency. hNPCs were either exposed to media supplemented with BDNF and PDGF to induce neurogenesis or with serum to induce astrogliogenesis. The extent of differentiation was determined at various time points (days 1, 3, 7, and 21) after exposure to the differentiation medium by assessing morphology and various markers.

### Immunocytochemistry

For immunocytochemistry, hNPCs were plated at a density of 20,000 cells per well in eight-well paranox chamber slides (Nunc, Kamstrupvej, Denmark). Cells were fixed with 4% paraformaldehyde for 20 min, washed three times with 1× PBS, blocked, and permeabilized by normal 10% goat serum (Vector Labs, Burlingame, CA, USA) containing 0.5% Triton X-100. Cells were incubated overnight with the following antibodies at 4 °C: DCX (Abcam, Cambridge, UK, 1:1000), SOX2 (Cell Signaling Technology, Denver, MA, USA, 1:200), MAP2 (Millipore, Billerica, MA, USA, 1:200), Nestin (Millipore, Billerica, MA, USA, 1:200), GFAP (Santa Cruz Biotechnology, 1:200), Tuj1 (Promega, Madison, WI, USA, 1:3000), and anti-Ki67 (Novacastra, Wetzlar, Germany, 1:1000). After incubation, cells were washed three times and incubated with fluorophore-tagged secondary antibody Alexa Fluor 488 and Alexa Fluor 594 (Invitrogen). Slides were mounted with DAPI containing Vectashield mounting media (Vector Labs). At least from five random fields, images were captured for each group using AxioImager.Z1 microscope (Carl Zeiss, Heidenheim, Germany). Numbers of cells were counted using ImageJ software, and neurite lengths were measured using the ImageJ plugin “Neurite Tracer”^43^.

### RNA isolation, RT-PCR, and gene expression analysis using qPCR

RNA was isolated from cells using Trizol reagent (Invitrogen, Eugene, USA). The cDNA was synthesized using BluePrint^TM^ 1st strand cDNA synthesis kit (Takara Bio Inc.), according to the manufacturer’s protocol. qPCR with specific primer pairs for *BASP1-AS1*, *BASP1*, and various relevant genes was used to detect their respective expression (a list of primer pairs is available with Supplementary S1). This was done by using SYBR^®^ Green PCR master mix (Applied Biosystem, USA) and Rotor-Gene Q (Qiagen, Germany).

### siRNA transfection

siRNA-mediated knockdown was employed to downregulate cellular expression of *BASP1*, *BASP1-AS1*, and *TCF12* in NPCs. Predesigned siRNA (Invitrogen) was used at a concentration of 40 pmoles. Cells were seeded at 80% confluency in 12-well format. According to the manufacturer’s protocol, knockdown was carried out using RNAimax (Invitrogen). For the control group, scrambled siRNA (Sigma) was used at a concentration of 40 pmoles. Transfection was carried out for 24 h, and samples were processed as per experimental requirement. To check the effect of *BASP1-AS1*-KD on the third day of differentiation, we performed knockdown on days 1 and 2 using siRNA.

### Chromatin isolation by RNA purification (ChIRP)

For this method, biotinylated probes were designed against *BASP1-AS1* transcript and the RT-qPCR primers for mapping genomic loci of *BASP1* gene that are interacting with the *BASP1-AS1*. We designed the antisense oligo biotinylated probes (listed in Supplementary S1) using the online probe designer singlemoleculefish.com. In total, 31 probes were designed in the frequency of one probe per 100 bp, and the probes were encompassed in the entire *BASP1-AS1* transcript. Next, we designed 14 primer pairs for the *BASP1* gene. These primer pairs map to exons 1 and 2, and to 1500 bp upstream and 1000 bp downstream of the gene. *BASP1-AS1* RNA pulldown was done for each using streptavidin-coated magnetic beads and followed by real-time PCR to identify enriched genomic loci of the *BASP1* gene.

ChIRP was performed as previously described^26^. NPCs on day 1 of differentiation were cross-linked with 1% glutaraldehyde at room temperature, and the reaction was quenched by 1/10th volume of 1.25 M glycine at room temperature after 10 min. The cell pellet was resuspended in lysis buffer (10× the mass of the pellet) consisting of 50 mM Tris-Cl (pH 7.0), 10 mM EDTA, 1% SDS, protease inhibitor, and RNase inhibitor (Superase-in, Ambion) and subjected to lysis by sonication in bioruptor at maximum setting with 30 s ON and 60 s OFF pulse intervals for 30 cycles. Chromatin was hybridized with a mixture of 31 biotinylated probes in a hybridization buffer consisting of 750 mM NaCl, 1% SDS, 50 mM Tris-Cl (pH 7.0), 1 mM EDTA, 15% formamide, a protease inhibitor, PMSF, and RNase inhibitor at 37 °C for 4 h (sequences for biotin-labeled probes are provided in Supplementary S1). The biotin–probe–chromatin complexes were pulled down with streptavidin magnetic beads and washed with 1 ml of wash buffer five times at 37 °C with shaking for 5 min. The last bit of wash buffer (2× SSC, 0.5% SDS, and add PMSF fresh) was removed completely with a sharp 10-μl pipette tip. Both RNA and DNA were eluted from these beads by different elution protocols according to the required downstream assays.

### Nuclear and cytoplasmic fractionation

Approximately 2 million cells were scraped with 500 μl of nuclear fractionation buffer (sucrose 1.2 M, HEPES 20 mM, KCl 10 mM, MgCl_2_ 2 mM, EDTA 1 mM, and EGTA 1 mM), and the cell suspension was passed through a 25-gauge needle ten times using a 1-ml syringe, and the cell suspension was incubated on ice for 20 min. Nuclei were then pelleted at 3000 rpm for 5 min, and the supernatant was stored for cytoplasmic RNA isolation. Pelleted nuclei were washed with 500 μl of fractionation buffer and passed again through a 25-gauge syringe needle ten times and centrifuged again at 3000 rpm for 10 min. After discarding the supernatant, the pellet was used for RNA isolation. RNA was isolated by Trizol reagent (Invitrogen, Eugene, USA) according to the manufacturer’s instruction.

### In situ hybridization (ISH) of *BASP1-AS1*

A protocol for in situ detection of long noncoding RNA *BASP1-AS1* was adapted from Fatima et al.^22^. Formalin-fixed, paraffin-embedded sections were derived from the brain region containing the SVZ area of an autopsied 23-week-old human fetus, obtained from Human Brain Tissue Repository, National Institute of Mental Health and Neurosciences, Bangalore, India. Sections were deparaffinized, dehydrated, treated with DEPC for 1 min, washed thrice with 1× PBST, and then treated with proteinase K (Invitrogen, USA) for 15 min following three washes with 1× PBST. Brain sections were fixed using 4% paraformaldehyde for 10 min. Hybridization buffer was composed of 50% formamide, 500 µg/ml yeast RNA (Ambion, USA), and 10% dextran sulfate (Sigma-Aldrich, St. Louis, MO, USA) with 0.16 M EDC (Sigma-Aldrich, St. Louis, MO, USA). Fixed sections were prehybridized at 59 °C with hybridization buffer for 4 h in a humified chamber. Sections were incubated with 40 nM of LNA 3′ and 5′ DIG-labeled lncRNA probe (Exiqon, Vedbaek, Denmark) overnight at 59 °C in a humidified chamber. Sections were washed for 5 min 6 times with wash buffer (50% 2× SSC and 50% formamide) at hybridization temperature. After 2 h of blocking with 20% sheep serum (Abcam, Cambridge, UK), sections were incubated overnight with anti-DIG AP-labeled antibody (Abcam, Cambridge, UK). After the incubation, sections were washed six times with AP buffer composed of 100 mM Tris-HCL, pH 9.5, 50 mM MgCl_2_, 100 mM NaCl, and 0.1% Tween 20. NBT/BCIP solution (Roche, Mannheim, Germany) was diluted in AP buffer, and sections were incubated with diluted NBT/BCIP for 10 h in a dark chamber. Sections were rehydrated, washed in xylene, mounted in DPX, and photographed using Olympus BX51 (Tokyo, Japan).

### Chromatin immunoprecipitation (ChIP)

In total, 5 × 10^6^ NPC cells were fixed with 1% glutaraldehyde at room temperature for 10 min. The reaction was then quenched with using 1.25 M glycine. The cells were pelleted and washed with 1× PBS twice. The pellet was resuspended in 1× lysis buffer (50 mM HEPES-KOH, pH 7.5, 140 mM NaCl, 1 mM EDTA, 1% Triton X-100, 0.1% SDS, and 1 mM PMSF). In all, 50 μl of Dynabeads (Invitrogen, USA) were washed thrice in 1 ml of block solution (1× PBS and 0.5% BSA) using a magnetic strip. Dynabeads were resuspended in 750 μl of block solution, and 10 μg of TCF12 primary antibody (Novus Biologicals, USA) was added to the beads and incubated overnight at 4 °C on a rotator. The beads were washed thrice in 1 ml of block solution. The cell pellet resuspended in lysis buffer was sonicated using Bioruptor^®^ Plus sonication device (Diagenode, Belgium) with 30 s ON and 60 s OFF pulse intervals for 30 cycles and centrifuged at 20,000 *g* for 10 min at 4 °C. In total, 50 μl of the supernatant was kept to be used as input DNA. Calibrated beads were added to the remaining supernatant and incubated overnight at 4 °C on a rotator. Three different buffers were used to wash the beads: (1) IP1 (lysis buffer and 500 mM NaCl), (2) IP2 (10 mM Tris-HCl, 250 mM LiCl, 1 mM EDTA, 0.5% NP-40, and 0.5% sodium deoxycholate with a final pH 8.0), and (3) TE (10 mM TRIS, pH 7.4, and 1 mM EDTA). Beads complexes were washed with lysis buffer five times, and six times each with IP1, IP2, and TE, and centrifuged at 960 *g* for 3 min at 4 °C. For elution of DNA, 210 μl of elution buffer (TE and 1% SDS) was added to the beads, and incubated at 65 °C for 15 min in a water bath. Beads containing elution buffer were centrifuged at 16,000 *g* for 1 min at room temperature. In total, 200 μl of supernatant is further processed for DNA isolation as described in “CHIRP Method” section discussed above. Isolated DNA was used for detection of BASP1 chromatin interacting with TCF12 using qPCR. Sequences of the primers used are provided in Supplementary file S2.

### Statistical analysis

Data are represented as mean ± S.D. All experiments were carried out using three biological replicates. Significance of the comparison between scrambled and different treated groups was computed using Student’s *t* test. *P* < 0.05 was considered as minimum or statistically significant.

## Supplementary information

Supplementary Figures S1

Supplementary Figures S2

Supplementary Figures S3

Supplementary Figures S4

Supplementary Figures S5

Supplementary Figures S6

Supplementary Figures S7

Supplementary Figures S8

Supplementary Figures S9

Supplementary Figures S10

Supplementary S1

Supplementary S2
