# Supplementary figures and images for: Temporal transcriptome analysis of neuronal commitment reveals the preeminent role of the divergent lncRNA biotype and a critical candidate gene during differentiation
Source: Cell Death Discov. 2020 Apr 24;6:28. doi: 10.1038/s41420-020-0263-6 (PMC7181654; doi:10.1038/s41420-020-0263-6)

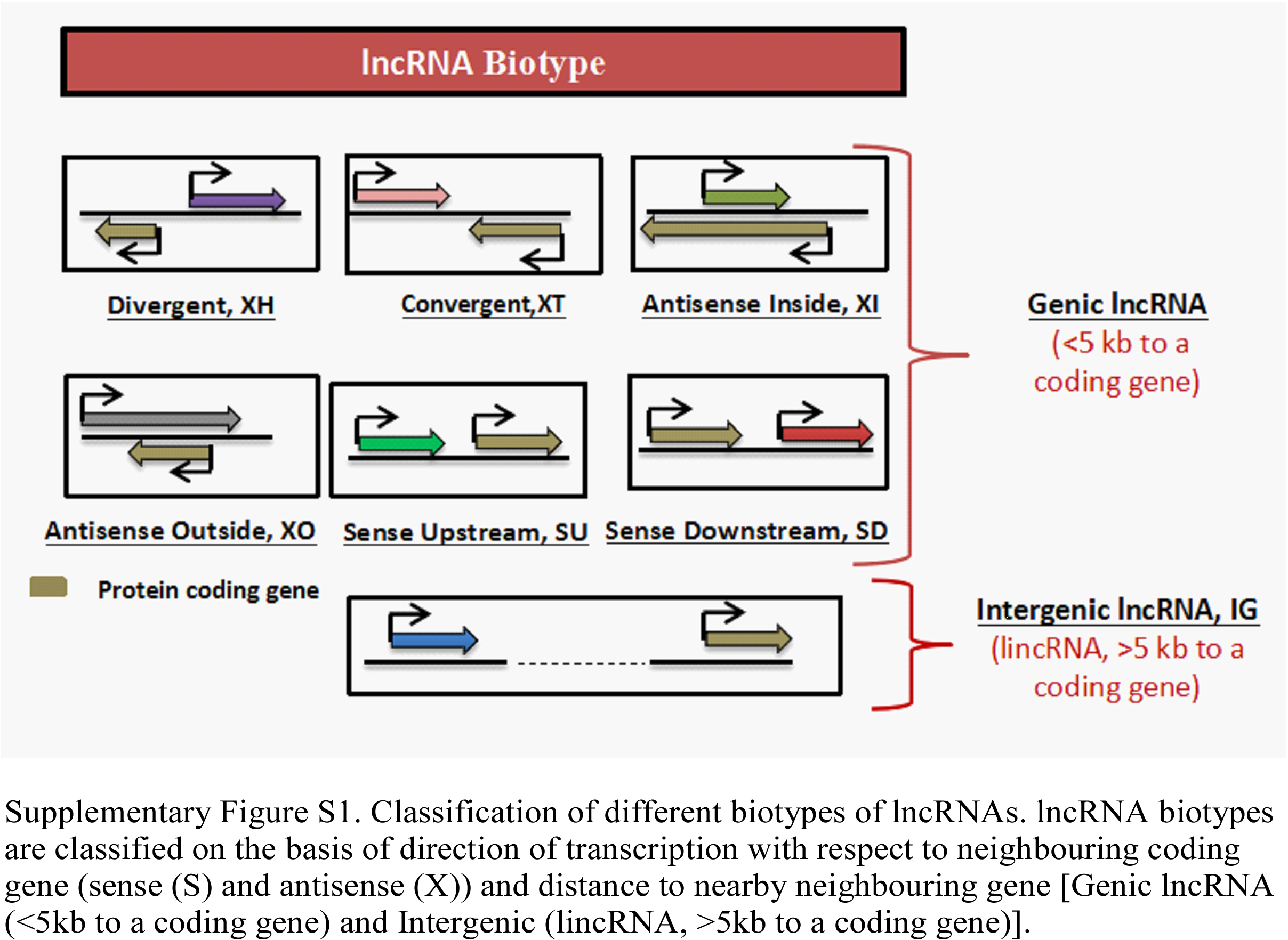

Supplement: Supplementary file 1 — Supplementary Figures S1 [file 41420_2020_263_MOESM1_ESM.tif]

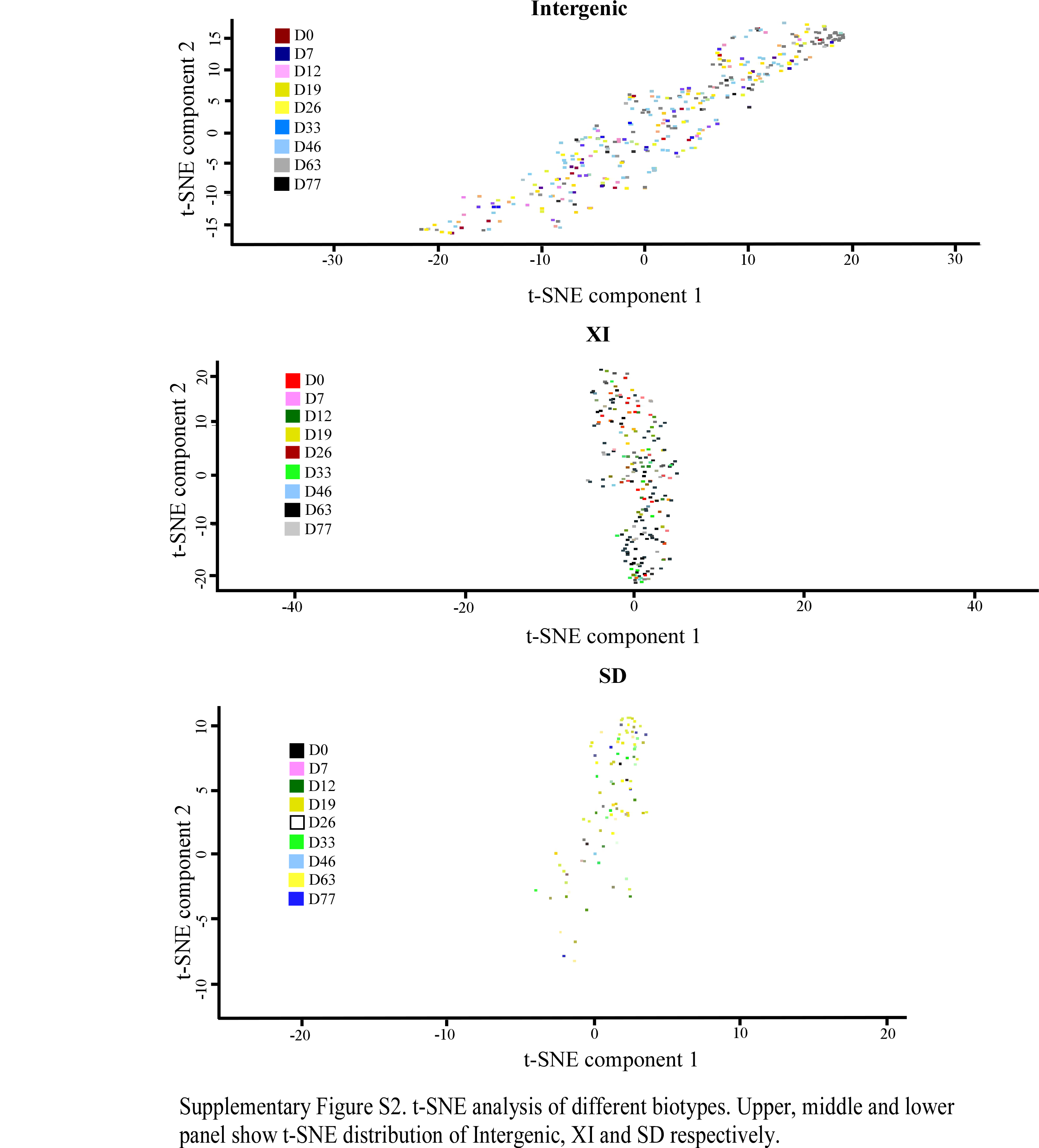

Supplement: Supplementary file 2 — Supplementary Figures S2 [file 41420_2020_263_MOESM2_ESM.tif]

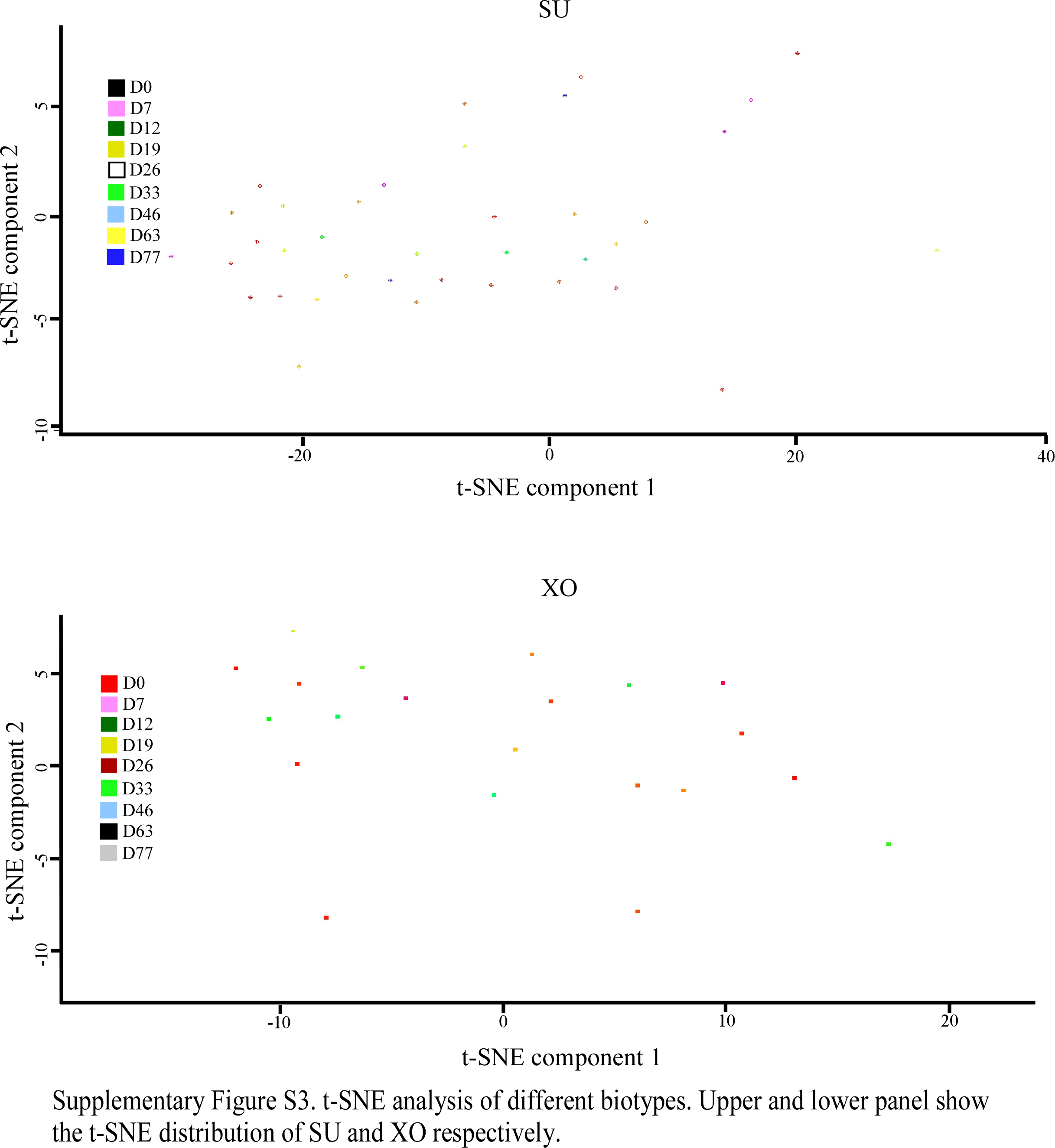

Supplement: Supplementary file 3 — Supplementary Figures S3 [file 41420_2020_263_MOESM3_ESM.tif]

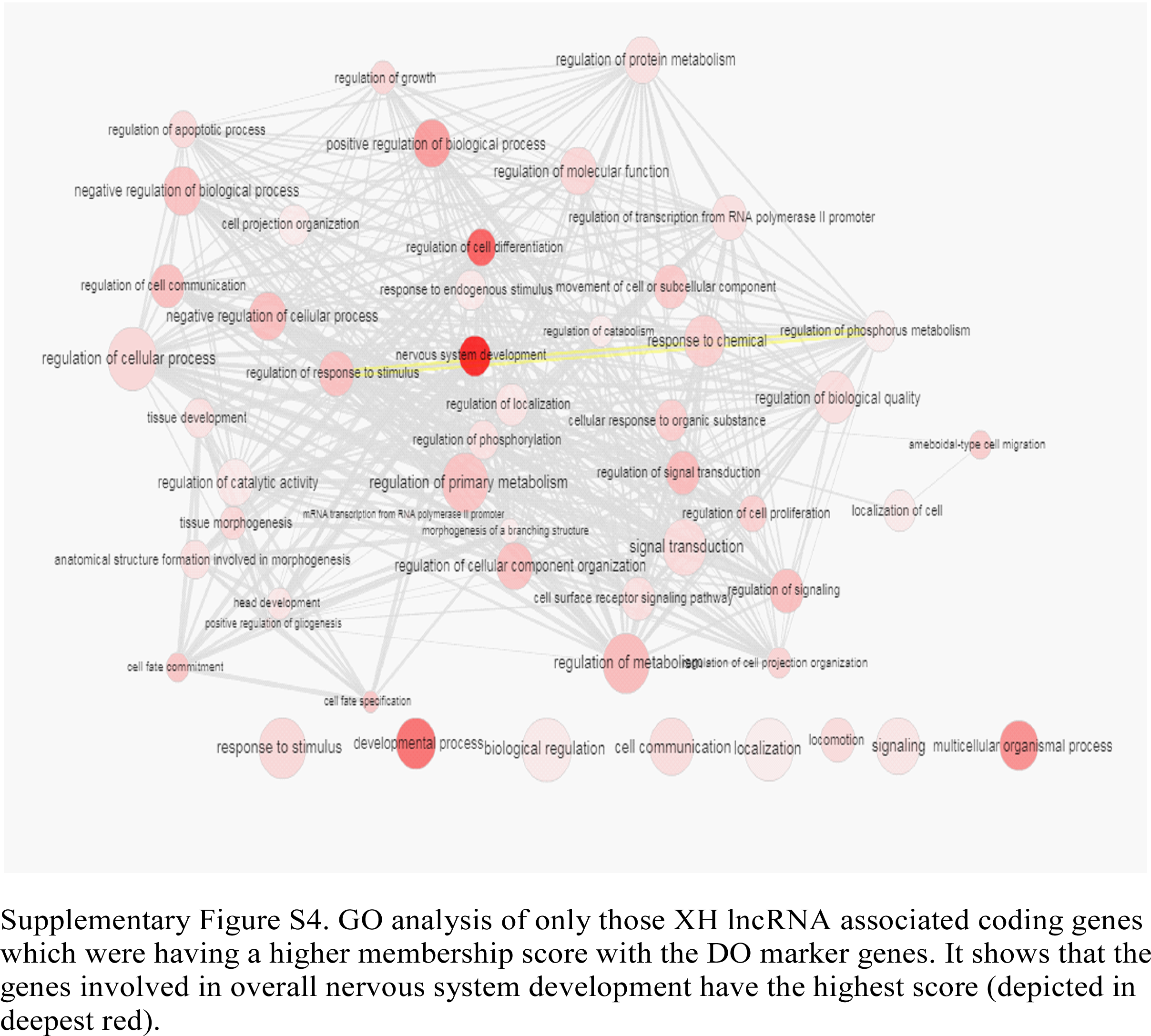

Supplement: Supplementary file 4 — Supplementary Figures S4 [file 41420_2020_263_MOESM4_ESM.tif]

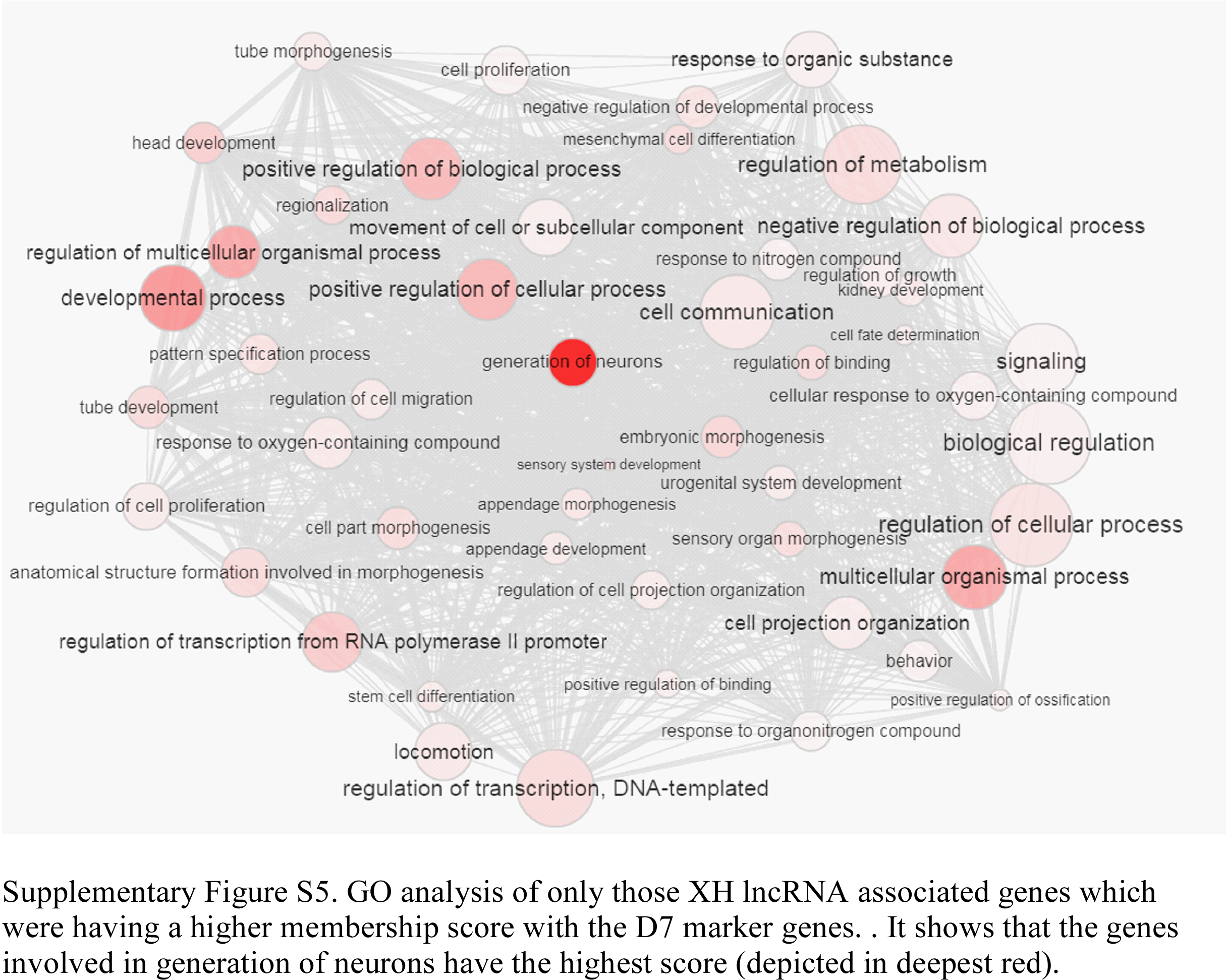

Supplement: Supplementary file 5 — Supplementary Figures S5 [file 41420_2020_263_MOESM5_ESM.tif]

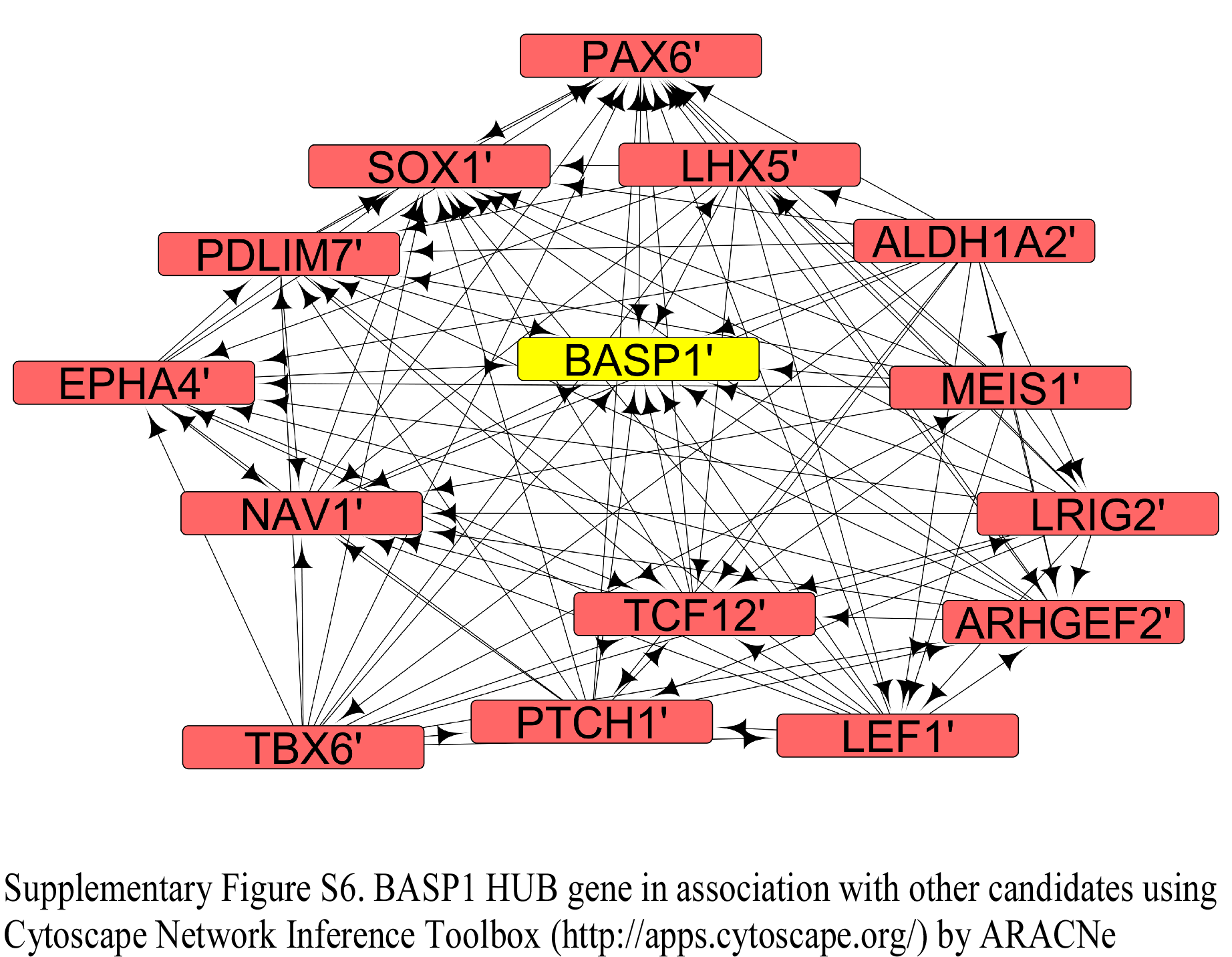

Supplement: Supplementary file 6 — Supplementary Figures S6 [file 41420_2020_263_MOESM6_ESM.tif]

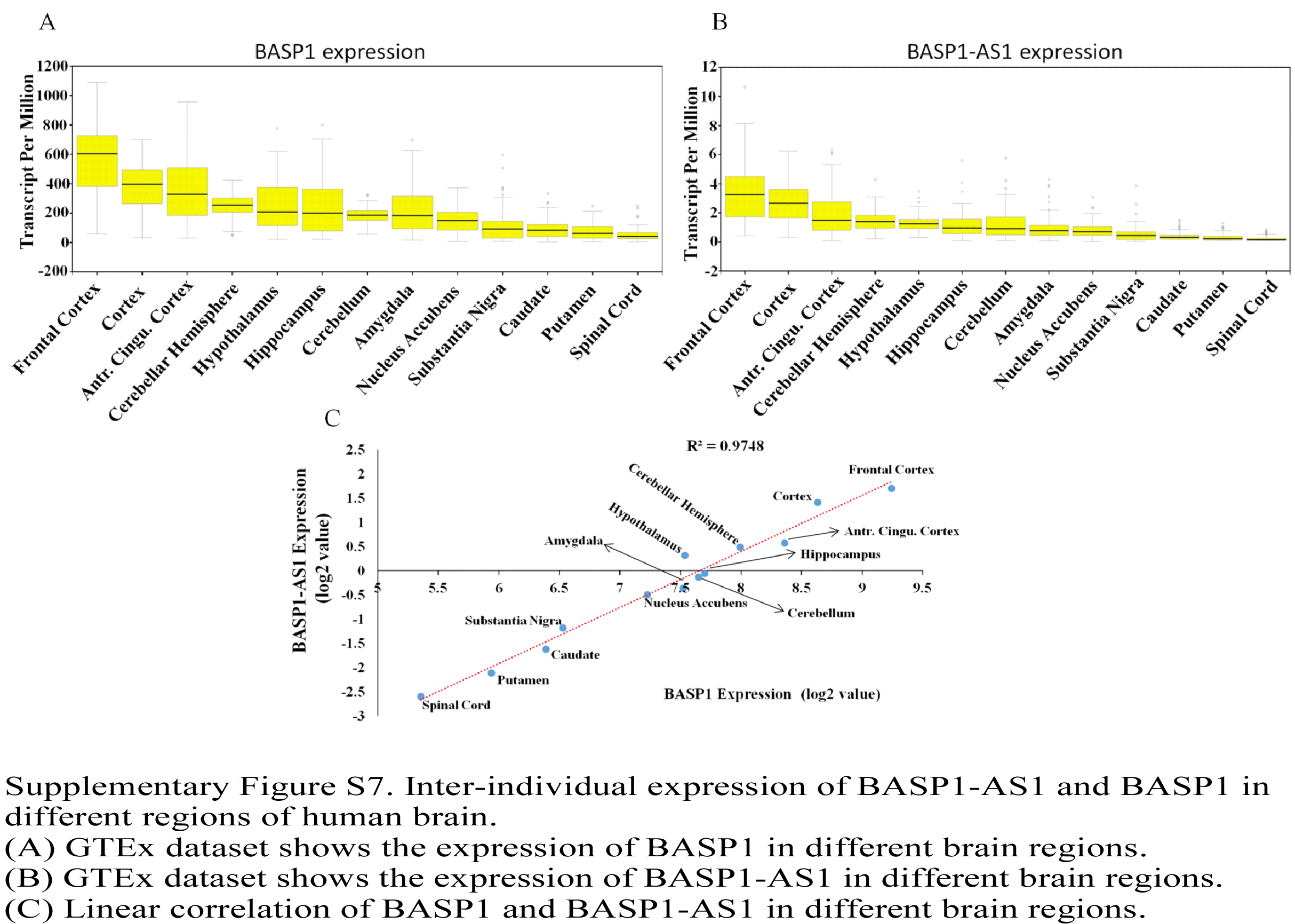

Supplement: Supplementary file 7 — Supplementary Figures S7 [file 41420_2020_263_MOESM7_ESM.tif]

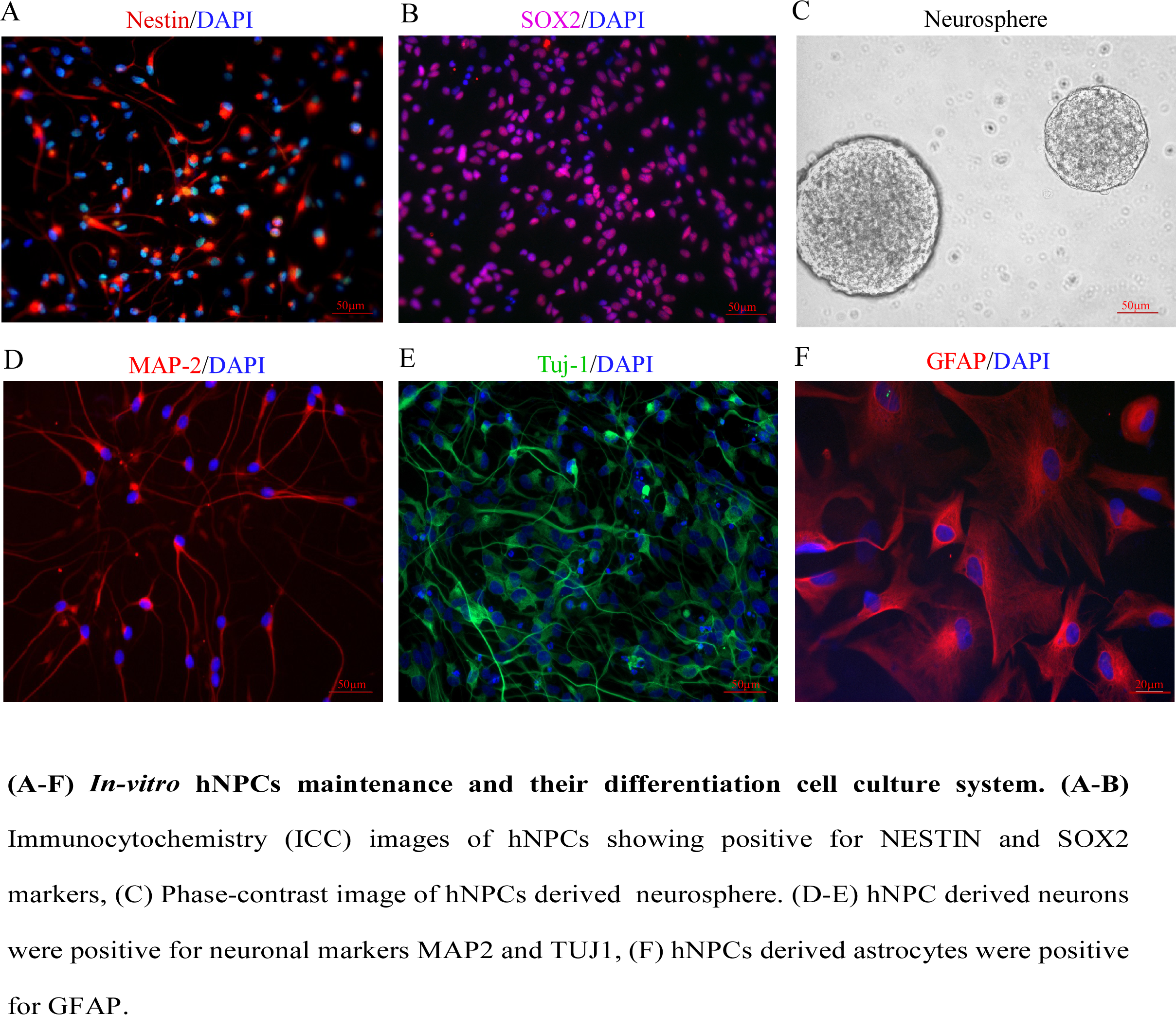

Supplement: Supplementary file 8 — Supplementary Figures S8 [file 41420_2020_263_MOESM8_ESM.tif]

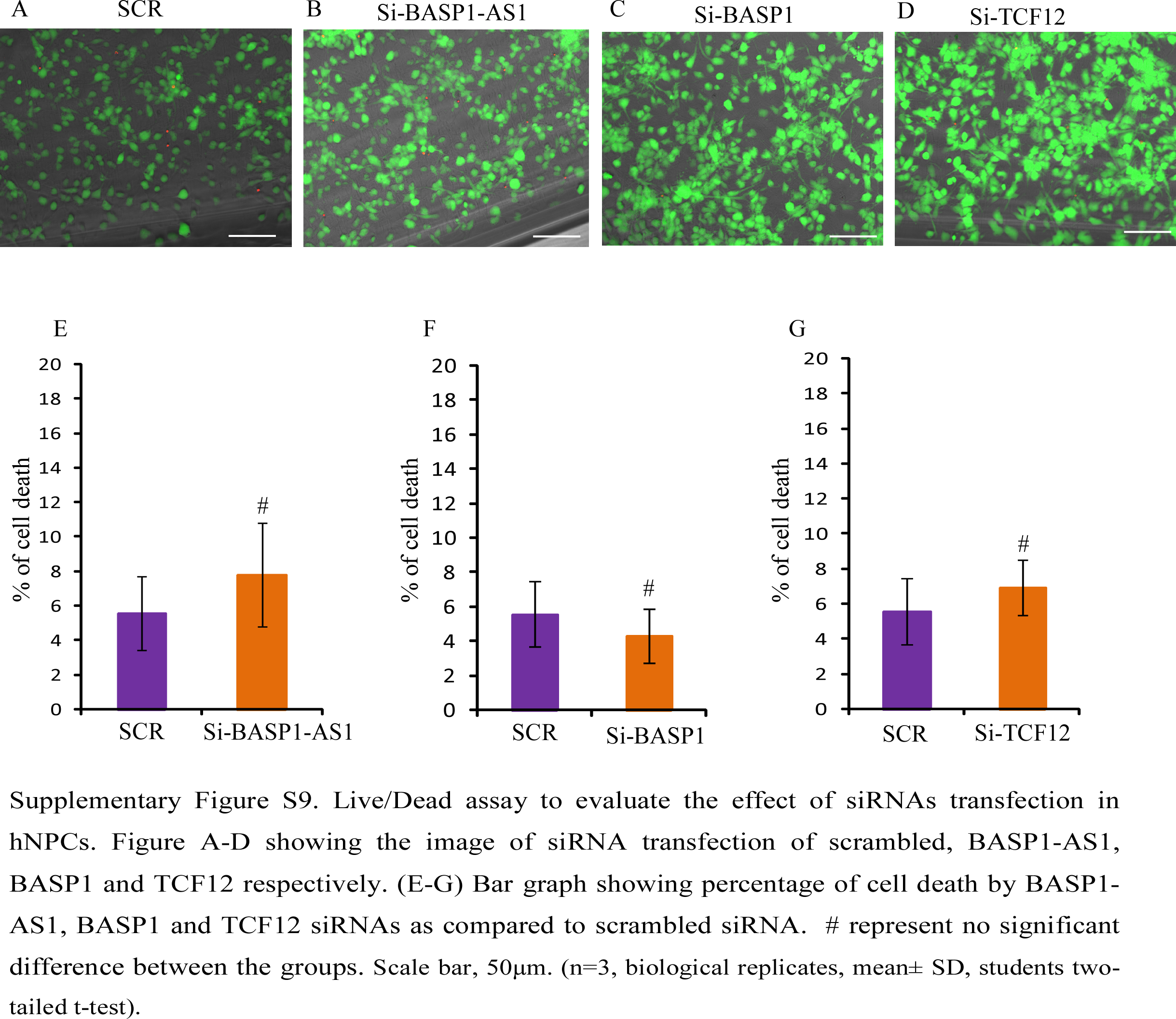

Supplement: Supplementary file 9 — Supplementary Figures S9 [file 41420_2020_263_MOESM9_ESM.tif]

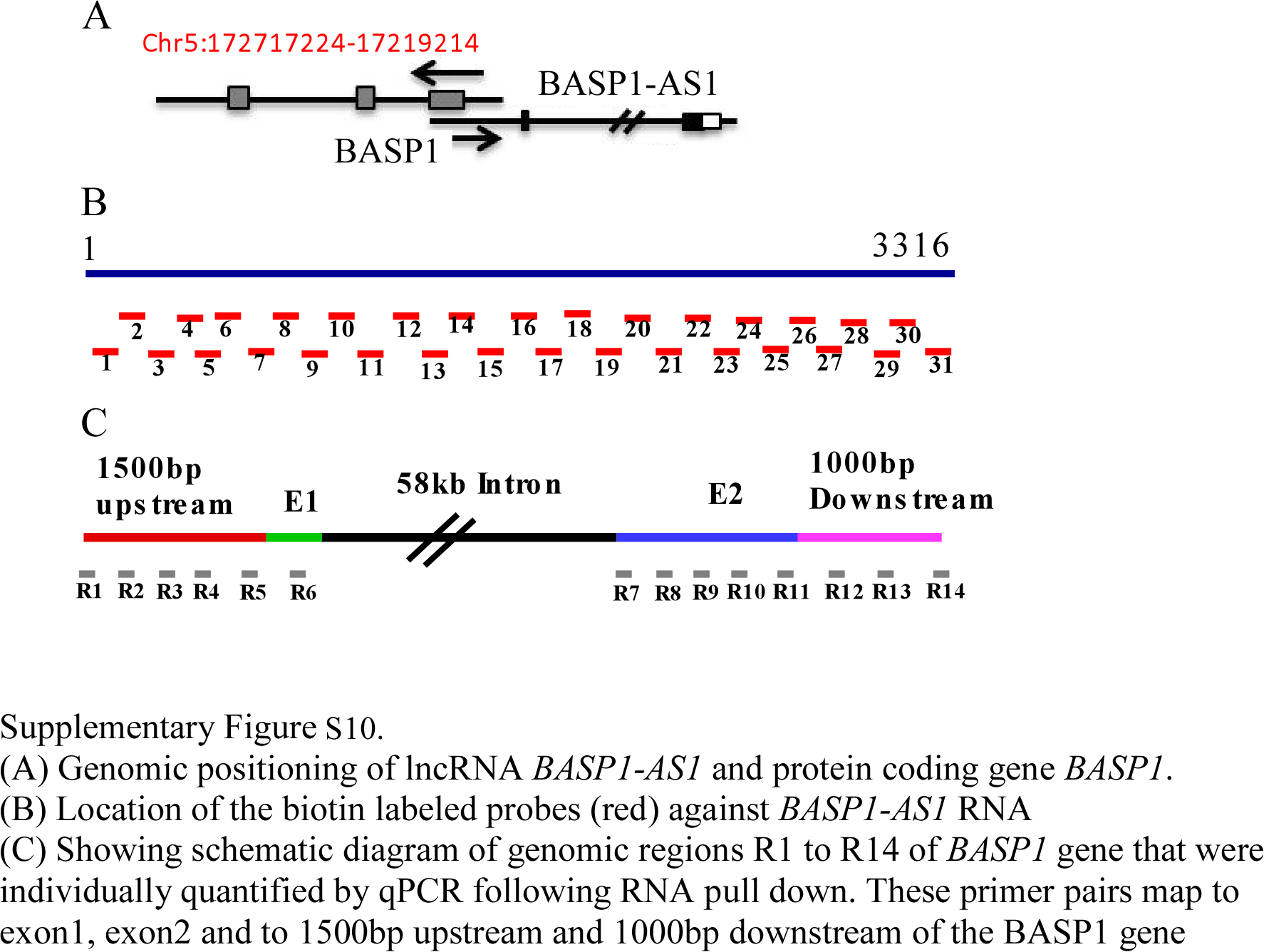

Supplement: Supplementary file 10 — Supplementary Figures S10 [file 41420_2020_263_MOESM10_ESM.tif]
